# Unraveling the phenotypic and genomic background of behavioral plasticity and temperament in North American Angus cattle

**DOI:** 10.1186/s12711-023-00777-3

**Published:** 2023-01-19

**Authors:** Amanda B. Alvarenga, Hinayah R. Oliveira, Simon P. Turner, Andre Garcia, Kelli J. Retallick, Stephen P. Miller, Luiz F. Brito

**Affiliations:** 1 Department of Animal Sciences, Purdue University, West Lafayette, IN USA; 2 Lactanet, Guelph, ON Canada; 3 Animal and Veterinary Sciences Department, Scotland’s Rural College, Edinburgh, UK; 4 American Angus Association, Angus Genetics Inc., Saint Joseph, MO USA; 5 AGBU, a joint venture of NSW Department of Primary Industries and University of New England, Armidale, 2351 Australia

## Abstract

**Background:**

Longitudinal records of temperament can be used for assessing behavioral plasticity, such as aptness to learn, memorize, or change behavioral responses based on affective state. In this study, we evaluated the phenotypic and genomic background of North American Angus cow temperament measured throughout their lifetime around the weaning season, including the development of a new indicator trait termed docility-based learning and behavioral plasticity. The analyses included 273,695 and 153,898 records for yearling (YT) and cow at weaning (CT) temperament, respectively, 723,248 animals in the pedigree, and 8784 genotyped animals. Both YT and CT were measured when the animal was loading into/exiting the chute. Moreover, CT was measured around the time in which the cow was separated from her calf. A random regression model fitting a first-order Legendre orthogonal polynomial was used to model the covariance structure of temperament and to assess the learning and behavioral plasticity (i.e., slope of the regression) of individual cows. This study provides, for the first time, a longitudinal perspective of the genetic and genomic mechanisms underlying temperament, learning, and behavioral plasticity in beef cattle.

**Results:**

CT measured across years is heritable (0.38–0.53). Positive and strong genetic correlations (0.91–1.00) were observed among all CT age-group pairs and between CT and YT (0.84). Over 90% of the candidate genes identified overlapped among CT age-groups and the estimated effect of genomic markers located within important candidate genes changed over time. A small but significant genetic component was observed for learning and behavioral plasticity (heritability = 0.02 ± 0.002). Various candidate genes were identified, revealing the polygenic nature of the traits evaluated. The pathways and candidate genes identified are associated with steroid and glucocorticoid hormones, development delay, cognitive development, and behavioral changes in cattle and other species.

**Conclusions:**

Cow temperament is highly heritable and repeatable. The changes in temperament can be genetically improved by selecting animals with favorable learning and behavioral plasticity (i.e., habituation). Furthermore, the environment explains a large part of the variation in learning and behavioral plasticity, leading to opportunities to also improve the overall temperament by refining management practices. Moreover, behavioral plasticity offers opportunities to improve the long-term animal and handler welfare through habituation.

**Supplementary Information:**

The online version contains supplementary material available at 10.1186/s12711-023-00777-3.

## Background

The increased interest in prolonging the lifespan of cattle hinges on a concern for longitudinal performance [1]. One way to enhance the long-term animal resilience in the herd is by improving temperament and stress-coping ability during handling, and consequently, providing economic benefits to the industry [2]. Likewise, handler welfare would be improved because fearful cattle are associated with an increased risk of handler injury [3]. In dairy cattle, temperamental cows tend to be frequently culled due to accidents as well as metabolic and digestive disorders compared to calmer cows [4].

Cattle temperament can be improved through management [3] and genetic and genomic selection, since moderate-to-high heritability estimates have been reported in the literature [5–7]. Phenotypic studies indicate that animals can get calmer with repeated positive interactions [3, 5, 8]. Parham et al. [3] concluded that producers may avoid early-in-life culling because the cows can habituate or become sensitized to handling, and consequently, change their behavioral responses over time. Habituation can be defined as decreased responsiveness to a stimulus as a result of repeated exposure, and sensitization is its antagonistic term (i.e., an increased responsiveness to a stimulus [9]). Habituation is often associated with adaptation [9]. Genomic studies in honeybees and humans indicate that certain genes can exert differential influence on behavioral response over time, suggesting that the expression of those genes can be regulated by adverse circumstances [10, 11].

Cognition comprises the ability to acquire, process, and use information, and relies on mechanisms including perception, learning and memory, and individual recognition [12]. Successful cognition should be apparent in adaptive behavioral responses, such as learning and behavioral plasticity. Studies have shown that cattle have the ability to learn and memorize their experiences and environments [3, 9, 13–16]. For instance, calves were able to discriminate locations based on probabilities of being rewarded or punished [17]. Besides, a hole-board test including 15 bottles (i.e., 11 empty and four baited bottles containing milk) assessed the memorization pattern of dairy calves over time based on the ratio of rewards by all visits and the number of bottle feeding locations visited [14]. Selection for learning and behavioral plasticity in livestock may provide an opportunity to improve long-term welfare and resilience for the animals and handler [13]. Furthermore, it can potentially promote better engagement and habituation of animals when handled by humans or when interacting with technology on modern farms.

A wide range (from 0.02 to 0.99) of heritability estimates have been reported for cognitive performance across many species, including humans, chimpanzee, rhesus macaque, domestic pig, rat, mouse, zebra finch, honey bees, guppy, and jungle fowl [18–22]. Similar to the genetic architecture of behavioral traits in livestock species [5, 6, 23, 24], learning and behavioral plasticity in humans follows a polygenic inheritance pattern [18, 20, 25].

To the best of our knowledge, no studies have evaluated the genomic factors that control temperament over time in cattle and the ability to learn and remember the experiences of previous handling as evidenced by rapid habituation. Our overarching goal was to characterize the changes in temperament (i.e., indicator trait of behavior) of North American Angus cattle across their lifetime from a genetic and genomic perspective. The specific objectives of this study were to (i) estimate (co)variance components for temperament over time, including heritability for cow-at-weaning temperament (CT) measured from 2 to 15 years of age and the genetic correlation among age-groups; (ii) perform a genome-wide association study (GWAS) of age-group cow temperament score (CT), followed by gene annotation analyses; (iii) investigate the changes in the effect of important genomic regions on cow temperament over time; (iv) evaluate if there is a heritable component on learning and behavioral plasticity as indicated by habituation in Angus cattle (measured by the slope of a random regression model based on a first order Legendre orthogonal polynomial); and, (v) identify genomic regions, candidate genes, and biological pathways influencing learning and behavioral plasticity in Angus cattle through single-step GWAS and functional annotation of candidate genes.

## Methods

### Data

All the datasets used were provided by Angus Genetics Inc. (AGI; Saint Joseph, MO, USA). For the initial investigation of this study, we considered two distinct datasets: yearling (YT) and cow temperament measured at weaning (CT). Temperament was subjectively scored by the handler on a 1-to-6 scale during handling in the chute. Score definitions are in Table 1, where score 1 represents a docile and score 6 represents a very aggressive animal, as described in Alvarenga et al. [5]. These scores and terminology represent the animal response to being handled and constrained in confined facilities. For the statistical genetic analyses, we clustered the scores 4, 5, and 6 into a single category due to their low incidence [5, 23].

**Table 1:** Temperament description and frequency of records for yearling temperament (YT) and cow temperament measured at weaning (CT) scores after the phenotypic quality control

Score	Description	YT	CT
1	“Docile- gentle and easily handled; stands and moves slowly during processing; exits chute calmly”	188,805 (70.12%)	82,739 (53.76%)
2	“Restless- quieter but may be stubborn during processing; may try to back out of chute; some flicking of tail; exits chute promptly”	63,189 (23.47%)	56,394 (36.64%)
3	“Nervous- typical temperament is manageable, but nervous and impatient; displays a moderate amount of struggling, movement, and tail flicking; repeated pushing; exits chute briskly”	14,985 (5.57%)	12,925 (8.40%)
4	“Flighty (wild)- jumpy and out of control, quivers, and struggles violently; may bellow and froth at the mouth; displays continuous tail flicking; defecates and urinates during processing; may jump when penned individually; exhibits long flight distance and exits chute wildly”	1928 (0.72%)	1325 (0.86%)
5	“Aggressive- like score 4, but added aggressive behavior, fearfulness, extreme agitation, and continuous movement, which may include jumping and bellowing while in the chute; exits chute frantically and may exhibit attack behavior when handled alone”	256 (0.10%)	452 (0.29%)
6	“Very aggressive- thrashes about or attacks wildly when confined in small, tight places; pronounced attack behavior”	85 (0.03%)	63 (0.04%)

This table represents the description and scoring guidelines reported in the Angus Journal Report (October 2007; [27]) and Beef Improvement Federation [28]

YT: yearling temperament, number of animals per score category; CT: cow at weaning temperament, number of records per score category

The percentage of animals/records with that scoring is given between parentheses

Yearling temperament was recorded on calves (males and females) from 320 to 440 days of age when animals were loaded into or left the chute. This dataset was previously described by Alvarenga et al. [5]. The phenotypic dataset was edited to keep a minimum of three records and variation (i.e., at least two different scores of YT) per contemporary group (CG) (described below). In total, 269,248 (153,387 males and 115,861 females) animals born between 1995 and 2018 with YT records were included in this study. The distribution of scores for YT is in Table 1.

**Fig. 1:**
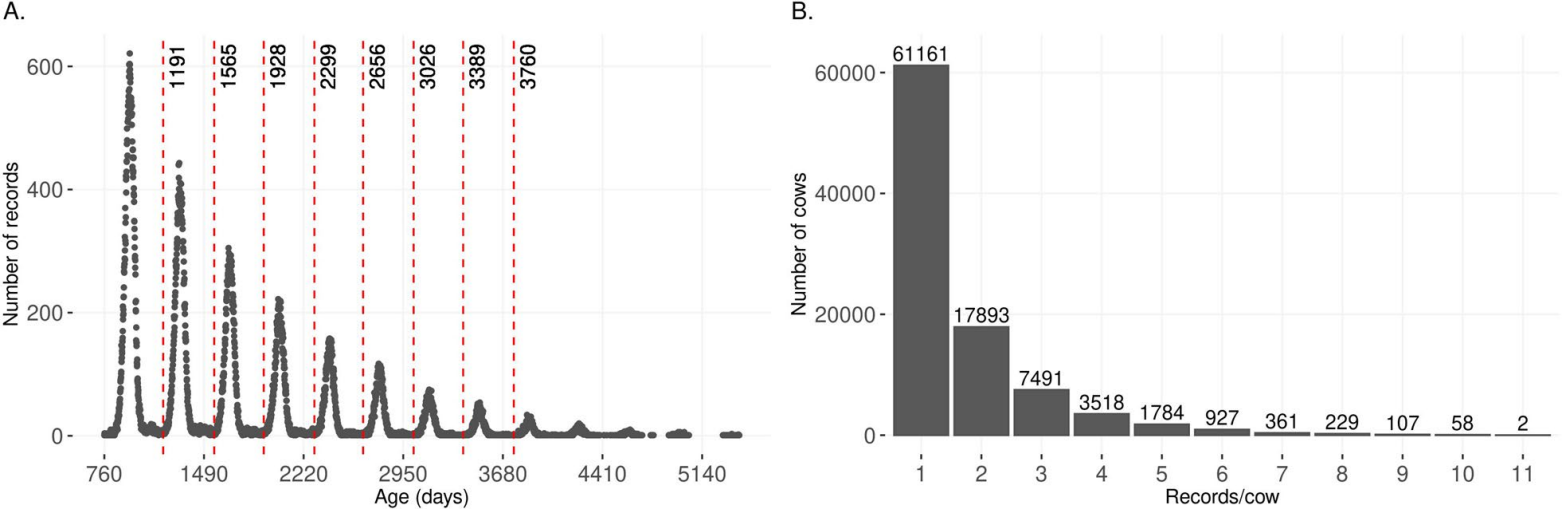
Distribution of the age in days for the cow at weaning temperament records (**a**) and the number of repeated records per cow (**b**). Vertical dashed-red lines are the thresholds used to cluster the records into nine age groups

Cow temperament measured at weaning was recorded on females around the weaning of their calves. The measurement was recorded on cows exiting the chute within a maximum interval of 45 days from the weaning event. CT was repeatedly measured across the cows’ lifetime. A data quality control for CT records included a minimum of three records and variation (i.e., at least two different scores of CT) per CG and a minimum interval between consecutive records of 268 days in the animal’s lifetime (threshold provided by AGI). We would not expect a cow to wean two calves within an interval smaller than 268 days, which is an extreme lower-bound value. After the quality control, 153,898 CT records for 93,531 cows born between 1978 and 2017 were used for this study. Data were collected from a minimum age of 760 days to a maximum of 5412 (~ 15 years) days (Fig. 1a). The records were clustered into nine age groups to facilitate the longitudinal statistical analyses. The vertical dashed-red lines in Fig. 1a represent the age group threshold, and detailed descriptive values are in Table 2. The number of records per animal ranged from one to 11 (Fig. 1b), with an average (standard deviation) of 1.7 (1.17) records and 30,586 animals with more than two records. The distribution of the scores for CT after quality control is in Table 1 while the distribution of scores per age-group interval is in Table 2. Phenotypic means across age-groups were statistically compared using the *t.test* function implemented in the R statistical software [26].

**Table 2:** Age group description and their frequencies and score distribution for yearling (YT, < 2 years) and cow at weaning (CT) temperament datasets

G	N	Age	Temperament scores (%)						Mean (SD)
			1	2	3	4	5	6	
< 2	269,248	375	70.12	23.47	5.57	0.72	0.10	0.03	1.37 (0.64)
3	47,839	760	56.00	35.53	7.49	0.73	0.22	0.03	1.54 (0.69)
4	33,579	1317	54.91	35.83	8.17	0.80	0.24	0.05	1.56 (0.71)
5	23,435	1682	53.71	36.48	8.60	0.85	0.32	0.04	1.58 (0.72)
6	16,817	2048	52.63	36.84	9.18	0.95	0.35	0.05	1.60 (0.73)
7	11,696	2414	51.84	37.59	8.90	1.15	0.46	0.07	1.61 (0.74)
8	8275	2778	50.10	39.70	8.65	1.11	0.37	0.06	1.62 (0.73)
9	5223	3144	49.21	39.71	9.78	1.00	0.31	0.00	1.63 (0.72)
10	3378	3511	47.51	39.93	10.89	1.01	0.56	0.09	1.67 (0.76)
> 10	3656	4128	46.17	41.52	10.94	1.04	0.33	0.00	1.68 (0.73)

G: age group; N: number of records; Age: average age in days; Mean (SD): mean (standard deviation) of the scores

There were 15,431 animals with measurements for both YT and CT. Furthermore, 31,679 calves recorded for YT had dams (21,656 dams) with CT measured during their weaning event. The pedigree dataset traced back four generations for the phenotyped animals, resulting in 723,248 individuals in the pedigree file.

### Statistical models

#### Models

The statistical model used for YT was previously defined by Alvarenga et al. [5]. In summary, it included conception type (i.e., embryo transferred or naturally conceived), age of dam (in years, categorical effect), and age of the animal at the measurement (i.e., linear covariate) as systematic effects; and, CG, animal additive genetic, and residual as random effects. CG was defined as a concatenation of birth month-year, birth herd, weaning month-year, weaning herd, creep-fed or not, temperament month-year, temperament herd, yearling group age (i.e., three groups based on their deviations from yearling age-interval: younger, yearling, or older), sex, and if for the animal ultrasound information was available or not (binary variable; zero for missing information [5]).

Several models including systematic effects and CG for CT (defined below) were tested to determine the optimal model. As a result, the optimal model included conception type (i.e., embryo transferred or naturally conceived—to capture potential preferential treatments of embryo transfer calves), age in days as a linear covariate, and herd-season-year at the trait scoring day as systematic effects; and CG, direct additive genetic, permanent environment, and residual as random effects. The CG was defined as weaning herd and month-year (i.e., herd and date when the cow was weaned in as a calf); if the cow received creep-feeding during the growing period; yearling herd, month-year, and age group (i.e., effects when the cow had around 1-year of age); if for the animal ultrasound information was available; and the herd and month-year at CT scoring. The completeness of information for both weaning and yearling variables was not required when doing data editing. In other words, animals could have unknown information for either of those variables to build the CG. The early-in-life management conditions of the cow (e.g., weaning, feeding system, yearling, and ultrasound measurements) were included in the CG when available because it can account for previous experiences that the animal had, which could potentially alter their behavior later in life. For instance, animals that are more frequently handled might have, on average, calmer behavior when exiting the chute than animals that are handled less frequently. CG was defined based on the statistical significance of the factors and our previous study on yearling temperament [5]. The statistical genetic analyses of this study comprised two steps: (1) pedigree-based (co)variance components estimations, and (2) single-step (ss)GWAS.

#### Methods

Threshold Bayesian methods (fitting a cumulative probit function) based on the Gibbs sampler and Markov chain Monte Carlo (MCMC) algorithm were used. (Co)variance components, breeding value prediction (EBV), and the proportion of the total additive genetic variance explained by single nucleotide polymorphisms (SNPs) were retrieved using the thrgibbs1f90 software [29]. Convergence of the components was verified using the Geweke, Heidelberger and Welch method, and visual criteria using the Bayesian Output Analysis package (*boa* [30]) implemented in the R software [26]. As an outcome of the threshold random regression model (RRM, described in more details in the next section), genomic estimated breeding values (GEBV) were obtained on the liability scale (not directly meaningful in the biological context compared to the original scale of the trait) and for the coefficients (i.e., intercept and slope-learning and behavioral plasticity). First, a transformation of the GEBV was performed from the liability to the probability scale to facilitate the interpretability of the GEBV as follows:

$${\mathrm{GEBV}}_{\mathrm{p}}=100*\mathrm{normalCDF}\left({t-\widehat{\mathrm{u}}}_{j}\right),$$

where ${\mathrm{GEBV}}_{\mathrm{p}}$ is the GEBV on the probability of being docile; $\mathrm{normalCDF}$ is the normal cumulative density function; $t$ is the threshold for which we calculated the cumulative probability, in this case, of being docile and its threshold was 0; and ${\widehat{\mathrm{u}}}_{j}$ is the average GEBV for the $j$ age group. To evaluate the SNP effects at each age group and potentially identify candidate regions controlling CT at specific ages, a second transformation of the GEBV was performed: from coefficients (i.e., intercept and slope) to GEBV for each age-group per animal:

$${\widehat{\mathbf{u}}}_{*}=\mathbf{T}\widehat{\mathbf{u}},$$

where ${\widehat{\mathbf{u}}}_{*}$ is a matrix containing $n$ rows (number of animals) and $j$ columns (age-groups); $\mathbf{T}$ is a matrix of independent covariates for the first-order Legendre orthogonal polynomial; and $\widehat{\mathbf{u}}$ is a vector of GEBV for the intercept and slope coefficients.

For the (co)variance components, a run with 500,000 samples, 250,000 burn-in, and thinning interval of 50 was performed. For the ssGWAS, 10,000 iterations, 5000 burn-in, and thinning interval of 5 was considered because the variance components were fixed based on previous analyses.

#### (Co)variance components estimation

We performed two (co)variance components analyses to answer different research questions, which are summarized in Table 3. First, we performed a random regression model (RRM) for CT (i.e., three to more than 10 age groups; Table 2; CT_RRM) fitting a Legendre orthogonal polynomial of first order. The main goal was to evaluate the covariance structure among temperament records across the years (age dependent) on cows and explore a learning and behavioral plasticity component as reflected by habituation to repeated handling (discussed below). CG, additive genetic, and permanent environmental effects were fitted as functions of age. The model used for CT_RRM was defined as follows:

**Table 3:** Summary of the (co)variance analyses performed in this study

Abbreviation	Methodology	Objective
CT_RRM	Single-trait random regression (CT dataset)	Evaluate the genetic covariance structure of temperament across cows’ lifetime and explore a potential learning and behavioral plasticity component reflected by habituation
YT_CT_MT	Two-trait model (YT vs. CT)	Explore the genetic covariance structure between yearling and cow at weaning temperament (based on a repeatability model)

CT: cow temperament measured at weaning; YT: yearling temperament

$$\mathbf{l}=\mathbf{X}\mathbf{b}+{\mathbf{X}}_{\mathbf{q}}\mathbf{q}+\mathbf{Z}\mathbf{u}+\mathbf{W}\mathbf{p}+\mathbf{e},$$

where $\mathbf{l}$ is the vector of CT on the liability scale; $\mathbf{b}$ is the vector of systematic effects (i.e., conception type) and coefficients of fixed regressions for age (in days) within herd-year-season at the trait scoring; $\mathbf{q}$ is the vector of random regression coefficients for the CG, $\mathbf{q}\sim \mathrm{N}(\boldsymbol{0},{\mathbf{R}}_{\mathbf{q}}\otimes \mathbf{I})$; $\mathbf{u}$ is the vector of random regression coefficients for the direct additive genetic effects, $\mathbf{u}\sim \mathrm{N}(\boldsymbol{0},{\mathbf{G}}_{\mathbf{u}}\otimes \mathbf{A})$; $\mathbf{p}$ is the vector of random regression coefficients for the permanent environmental effects, $\mathbf{p}\sim \mathrm{N}(\boldsymbol{0},{\mathbf{R}}_{\mathbf{p}}\otimes \mathbf{I})$; $\mathbf{e}$ is the random vector of residuals, $\mathbf{e}\sim \mathrm{N}(\boldsymbol{0},\mathbf{I}{\sigma }_{e}^{2})$. First-order Legendre orthogonal polynomials were used because we also aimed at evaluating a learning and behavioral plasticity component that would be feasible to interpret by considering the intercept and slope of the regression. A linear slope is an easily interpretable coefficient to evaluate changes overtime. $\mathbf{X}$, ${\mathbf{X}}_{\mathbf{q}}$, $\mathbf{Z}$, and $\mathbf{W}$ are the incidence matrices for $\mathbf{b}$, $\mathbf{q}$, $\mathbf{u}$, and $\mathbf{p}$; ${\mathbf{R}}_{\mathbf{q}}$, ${\mathbf{G}}_{\mathbf{u}}$, and ${\mathbf{R}}_{\mathbf{p}}$ are the CG, additive genetic, and permanent environmental variance component matrices; $\mathbf{I}$ and $\mathbf{A}$ are the identity and pedigree-based additive relationship matrices, respectively. Residuals were assumed to be homogeneous.

The (co)variance components for RRM were presented in two forms: coefficients (i.e., slope and intercept, in which the slope is termed as learning and behavioral plasticity) and over time (i.e., nine age groups). Heritability and repeatability estimates over time were calculated as:

$${\mathrm{h}}_{\mathrm{j}}^{2}=\frac{{\widehat{\upsigma }}_{{\mathrm{u}}_{\mathrm{j}}}^{2}}{{\widehat{\upsigma }}_{{\mathrm{u}}_{\mathrm{j}}}^{2}+ {\widehat{\upsigma }}_{{\mathrm{CG}}_{\mathrm{j}}}^{2}+ {\widehat{\upsigma }}_{{\mathrm{pe}}_{\mathrm{j}}}^{2}+ {\widehat{\upsigma }}_{\mathrm{e}}^{2}},$$

$$\mathrm{and }\,{\mathrm{rep}}_{\mathrm{j}}=\frac{{\widehat{\upsigma }}_{{\mathrm{u}}_{\mathrm{j}}}^{2}+{\widehat{\upsigma }}_{{\mathrm{pe}}_{\mathrm{j}}}^{2}}{{\widehat{\upsigma }}_{{\mathrm{u}}_{\mathrm{j}}}^{2}+{\widehat{\upsigma }}_{{\mathrm{CG}}_{\mathrm{j}}}^{2}+{\widehat{\upsigma }}_{{\mathrm{pe}}_{\mathrm{j}}}^{2}+ {\widehat{\upsigma }}_{\mathrm{e}}^{2}},$$

where ${\mathrm{h}}_{\mathrm{j}}^{2}$ and ${\mathrm{rep}}_{\mathrm{j}}$ are the heritability and repeatability estimated for the $j$ age group. The CG, direct additive genetic, and permanent environmental variance components for the $j$ age group are represented by ${\widehat{\upsigma }}_{{\mathrm{cg}}_{\mathrm{j}}}^{2}, {\widehat{\upsigma }}_{{\mathrm{u}}_{\mathrm{j}}}^{2}$, and ${\widehat{\upsigma }}_{{\mathrm{pe}}_{\mathrm{j}}}^{2}$, respectively, and they were calculated from the posterior mean of the (co)variance components estimated for the random regression coefficients of the CG, additive genetic, and permanent environment effects as:

$${\varvec{\upphi}}=\mathbf{T}{\mathbf{R}}_{\mathbf{q}}{\mathbf{T}}^{\mathrm{^{\boldsymbol{\prime}}}},$$

$${\varvec{\Sigma}}=\mathbf{T}{\mathbf{G}}_{\mathbf{u}}{\mathbf{T}}^{\mathrm{^{\boldsymbol{\prime}}}},$$

$$\mathrm{and}\,{\varvec{\uptheta}}=\mathbf{T}{\mathbf{R}}_{\mathbf{p}}{\mathbf{T}}^{\mathbf{^{\boldsymbol{\prime}}}},$$

where ${\varvec{\upphi}}$, ${\varvec{\Sigma}}$ and ${\varvec{\uptheta}}$ are the contemporary group, additive genetic, and permanent environmental (co)variance matrices for the age groups; and $\mathbf{T}$ is a matrix of independent covariates for the first-order Legendre orthogonal polynomial representing the different age groups. Then, we fitted a bivariate model (YT and CT), in which for CT we used a repeatability model instead of a RRM as previously described. The models fitted were:

$${\mathbf{l}}_{\mathbf{Y}\mathbf{T}}=\mathbf{X}\mathbf{b}+{\mathbf{X}}_{\mathbf{q}}\mathbf{q}+\mathbf{Z}\mathbf{u}+\mathbf{e},$$

$${\mathbf{l}}_{\mathbf{C}\mathbf{T}}=\mathbf{X}\mathbf{b}+{\mathbf{X}}_{\mathbf{q}}\mathbf{q}+\mathbf{Z}\mathbf{u}+\mathbf{W}\mathbf{p}+\mathbf{e},$$

where ${\mathbf{l}}_{\mathbf{Y}\mathbf{T}}$ and ${\mathbf{l}}_{\mathbf{C}\mathbf{T}}$ are the vectors of YT and CT on the liability scale; $\mathbf{b}$ is the vector of systematic effects, including age (as a covariate for both models), conception type (YT and CT models), and age of dam (years, YT); $\mathbf{q}$ is the vector of random CG; $\mathbf{u}$ is the vector of random direct additive genetic effects; $\mathbf{p}$ is the vector of permanent environmental effects; $\mathbf{e}$ is the vector of residual random effects; and $\mathbf{X}$, ${\mathbf{X}}_{\mathbf{q}}$, $\mathbf{Z}$, and $\mathbf{W}$ are the incidences matrices for $\mathbf{b}$, $\mathbf{q}$, $\mathbf{u}$, and $\mathbf{p}$, respectively.

#### Single-step genome-wide association studies

In total, 8784 genotyped animals were used for the ssGWAS for the CT based on the RRM. Of the 8784 genotyped animals, 5751 had their own CT records, 522 and 1795 were sires and dams of animals with CT phenotypes, respectively, and 716 were grandparents and/or great-grandparents of cows with CT records. These animals were genotyped with various SNP arrays as part of ongoing commercial genotyping activities for genetic evaluation purposes, resulting in a SNP set of 54,609 SNPs, as described in detail by Alvarenga et al. [5]. The genomic coordinates were based on the ARS-UCD1.2 bovine genome assembly [31, 32]. Genome-wide association results for YT were retrieved from Alvarenga et al. [5].

Genomic quality control procedures were applied using the preGSf90 software [33] and removed genotyped individuals with a call rate lower than 90% and pedigree errors. SNP genotypes with a call rate lower than 90%, a minor allele frequency (MAF) lower than 0.01, a deviation of the heterozygous genotype from Hardy Weinberg equilibrium higher than 0.15, and non-autosomal chromosomes were also removed. In total, 8748 genotyped animals and 41,376 SNPs were kept for further analyses.

The ssGWAS [34] method was used, in which the pedigree-based matrix tracking back four generations from the animals with phenotype information ($\mathbf{A}$, 282,170 animals) and SNP-based relationship matrix ($\mathbf{G}$) were combined into the $\mathbf{H}$ matrix [34–37]. The GEBV were obtained using the thrgibbsf190 software [29] and previously calculated variance components. The GEBV were back-solved to the SNP effects. In brief, the back-solving process can be described as:

$$\widehat{{\mathbf{u}}_{\mathbf{c}}}={\mathbf{M}}{^{{\mathbf{{\prime}}}}{{\lfloor\mathbf{M}{\mathbf{M}}{\mathbf{^{\prime}}}\rfloor}^{-1}\widehat{{\mathbf{G}\mathbf{E}\mathbf{B}\mathbf{V}}_{{\varvec{c}}}}}},$$

where $\widehat{{\mathbf{u}}_{{\varvec{c}}}}$ is the vector of SNP solutions for the $c$th random regression coefficient (intercept or slope); $\mathbf{M}$ is the matrix of genotypes coded as − 1, 0, and 1, representing *aa*, *Aa*, and *AA*, respectively, and $\widehat{{\mathbf{G}\mathbf{E}\mathbf{B}\mathbf{V}}_{{\varvec{c}}}}$ is the vector of GEBV for the $c$th random regression coefficient from all genotyped animals. The SNP solutions for both coefficients of the same SNP $k$ were combined into a vector and used to estimate the SNP effects for all age groups as $\widehat{{\mathbf{S}\mathbf{N}\mathbf{P}}_{{\varvec{k}}}}=\mathbf{T}\widehat{{\mathbf{u}}_{{\varvec{k}}}}$ where $\widehat{{\mathbf{S}\mathbf{N}\mathbf{P}}_{{\varvec{k}}}}$ is the vector containing the SNP effects estimated for each age group of the $k$th SNP, $\mathbf{T}$ is a matrix of covariates for the first-order Legendre orthogonal polynomial, and $\widehat{{\mathbf{u}}_{{\varvec{k}}}}$ is the vector of SNP solutions for both random regression coefficients related to the $k$th SNP. After obtaining the SNP effects for each coefficient, sliding windows of five SNPs were created (as done in Alvarenga et al. [5]) and the effects of those SNPs were summed up. The variance explained by each genomic window was calculated as proposed by Strandén and Garrick [38].

Quantitative trait loci (QTL) curated in the Cattle QTL Database (Cattle QTLdb [39] www.animalgenome.org; accessed date: 17 March 2022) and located within the selected genomic windows were identified. Gene annotation information was retrieved from Ensembl using the *biomaRt* R package [26, 40]. Functional annotation was performed in terms of Gene Ontology (GO) biological processes (GO_BP [41]) and metabolic pathways of the Kyoto Encyclopedia of Genes and Genomes (KEGG [42]) available in the DAVID database (david.ncifcrf.gov/tools.jsp [43]; access date: 19 March 2022).

## Results

### Phenotypic description of yearling and cow temperament measured at weaning

**Fig. 2:**
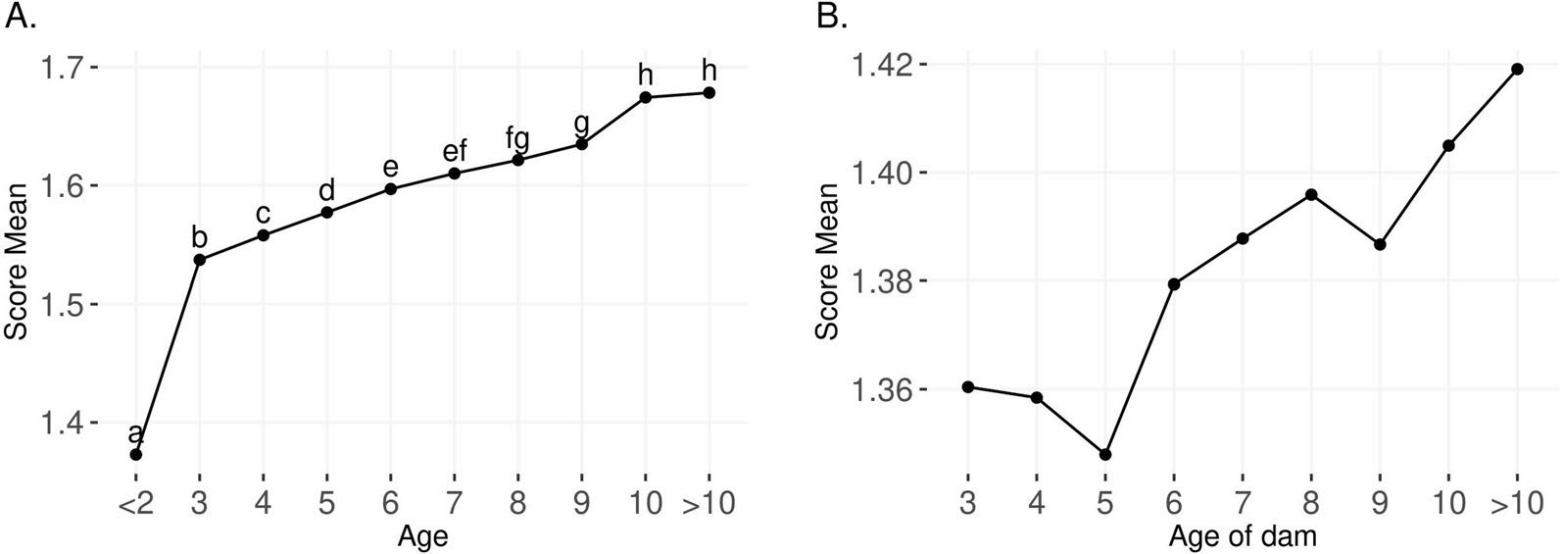
Phenotypic average of temperament across age groups and their significance (**a**) and the phenotypic average of yearling temperament grouped by age of dam (**b**)

Older animal groups had a significantly higher temperament (flightier) score than younger animal groups based on a test of means (Fig. 2a and Table 2). Animals younger than 2 years had a phenotypic mean YT of 1.38 (standard deviation equal to 0.64) and animals older than 10-years-old had a mean of 1.67 (0.76; Table 2). Although the difference is small, older dams tended to raise flightier calves (mean equal to 1.42) compared to younger dams (mean equal to 1.36; Fig. 2b). Finally, the phenotypic Pearson correlation between CT and YT of their progeny was 0.36.

### (Co)variance components of cow temperament measured at weaning

#### Temperament over time

**Fig. 3:**
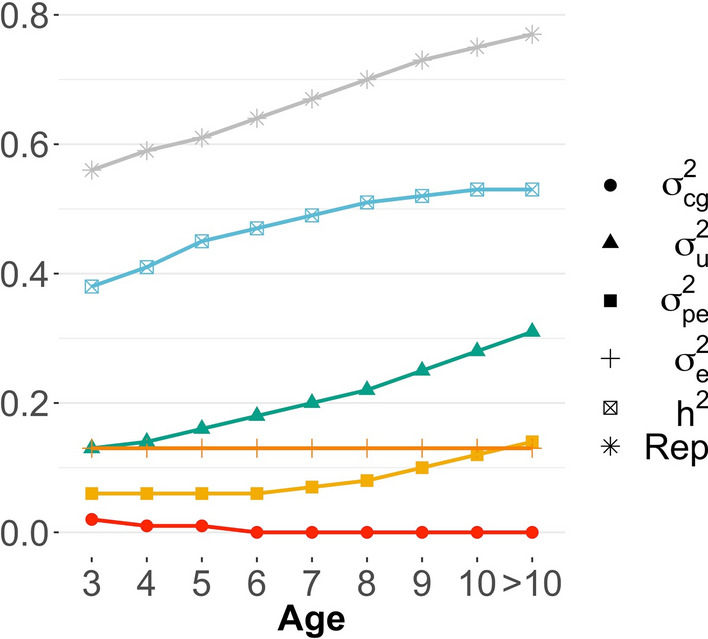
Variance, heritability, and repeatability estimates across the years for cow at weaning temperament. ${\upsigma }_{\mathrm{cg}}^{2}$ is the contemporary group variance; ${\upsigma }_{\mathrm{u}}^{2}$ is the additive genetic variance; ${\upsigma }_{\mathrm{pe}}^{2}$ is the permanent environmental variance; ${\upsigma }_{\mathrm{e}}^{2}$ is the residual variance; ${\mathrm{h}}^{2}$ is the heritability; and Rep is the repeatability

**Fig. 4:**
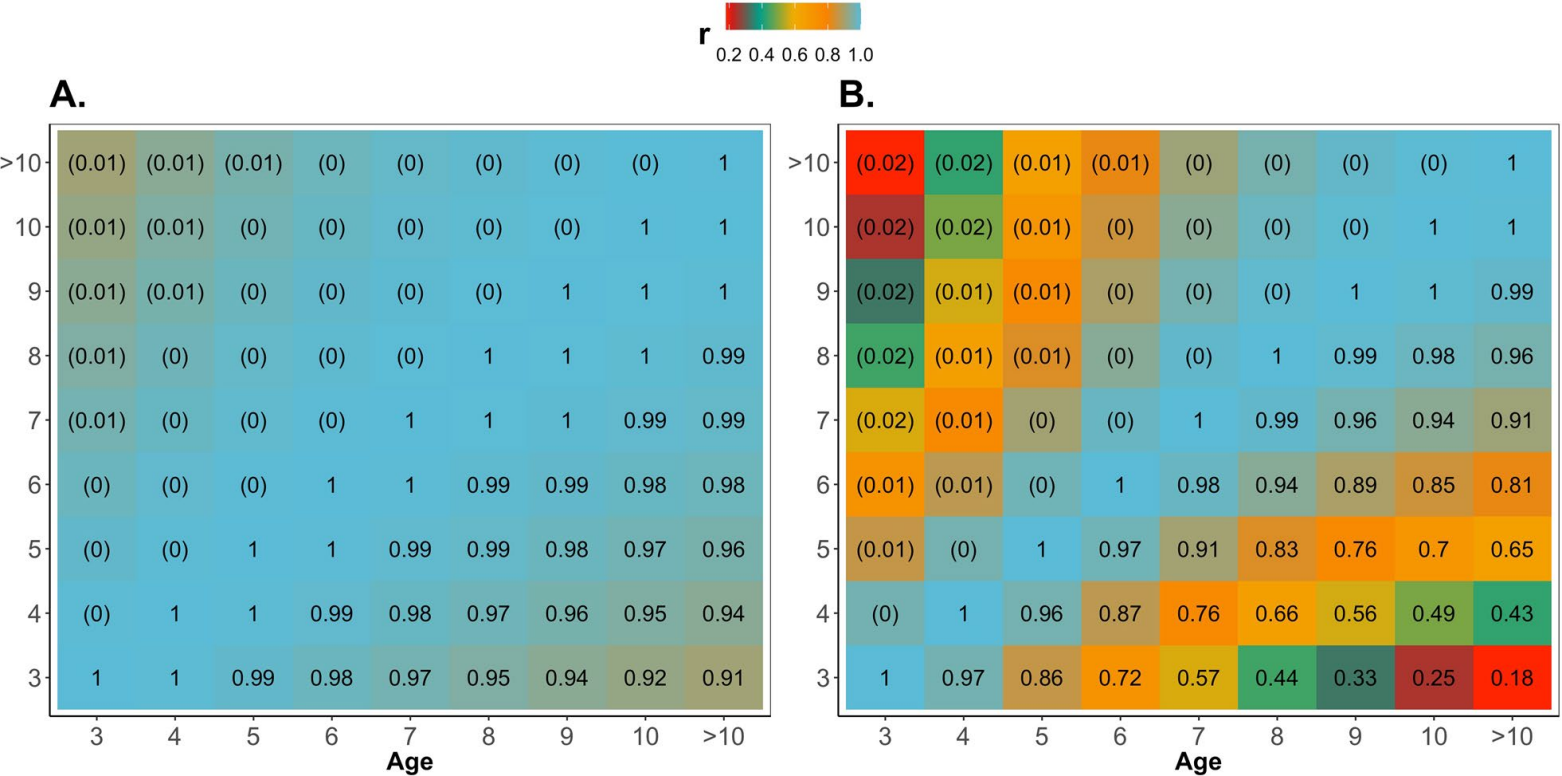
Additive genetic (**a**) and phenotypic (**b**) correlations among pairs of age group for temperament measured on cows at weaning. Lower diagonal: correlations; upper diagonal: values between parentheses are the standard deviations for the correlations

On the one hand, the variance of the CG slightly decreased with age (circle-solid red line in Fig. 3). On the other hand, the additive genetic and permanent environmental variances increased over time (triangle-green and square-yellow lines, respectively; Fig. 3). Residual variances were assumed to be homogeneous over time and were equal to 0.13 (0.01). Heritability and repeatability increased over time from 0.38 (0.02) to 0.53 (0.03) and from 0.56 (0.01) to 0.77 (0.01), respectively (Fig. 3). The (co)variance components for all age groups are in Additional file 1: Table S1. High genetic correlations were observed among all pairs of age for CT, ranging from 0.91 to 1.00 (down-diagonal Fig. 4a). The phenotypic correlation decreased as the gap in age increased (from 1.00 to 0.18; down-diagonal Fig. 4b). In the upper diagonal of Fig. 4, values within parentheses, are the standard deviations from the posterior distribution for the correlations.

#### Learning and behavioral plasticity

The average CT and learning and behavioral plasticity components were represented by the intercept and slope coefficients of the random regression model based on a first-order Legendre orthogonal polynomial (CT_RRM), respectively. The coefficients and (co)variances for the average CT and learning and behavioral plasticity are in Table 4. The learning and behavioral plasticity component explained 3.3% of the total additive genetic variation of cow temperament measured at weaning. A low heritability estimate (0.016; Table 4) was observed for the learning and behavioral plasticity; however, it is significantly different from 0 (highest probability density: 0.012–0.020). Permanent environment effects had a larger impact on learning and behavioral plasticity with a variance 1.6 times greater than the direct additive genetic variance, compared to average CT, in which the additive genetic variance was 2.8 times greater than the permanent environment variance (Table 4).

**Table 4:** (Co)variance components for the average cow temperament measured at weaning (average CT – intercept) and learning and behavioral plasticity (LBP – slope)

Component	Average CT	LBP
Contemporary group variance (${\sigma }_{CG}^{2}$)	0.004 (0.001)	0.005 (0.001)
Additive genetic variance (${\sigma }_{u}^{2}$)	0.396 (0.019)	0.013 (0.002)
Permanent environment variance (${\sigma }_{pe}^{2}$)	0.143 (0.013)	0.021 (0.003)
Residual variance (${\sigma }_{e}^{2}$)^a^	0.129 (0.008)	
Heritability (${h}^{2}$)	0.505 (0.018)	0.016 (0.002)
Repeatability	0.688 (0.007)	0.043 (0.004)
Genetic correlation	0.744 (0.046)	
Phenotypic correlation	0.219 (0.013)	

Average CT: overall average cow at weaning temperament, the intercept of the random regression model; LBP: learning and behavioral plasticity, the slope of the random regression model

^a^Common residual variance for intercept and slope because homogenous residuals were fitted for all age groups

**Fig. 5:**
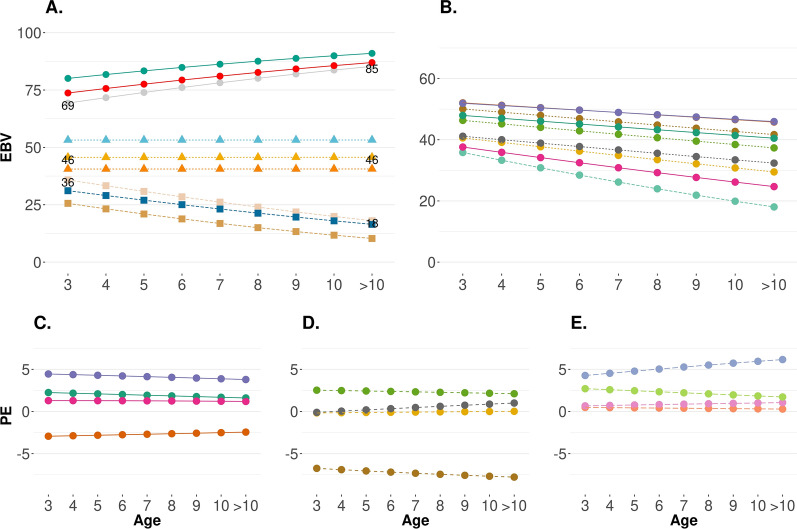
Genetic and permanent environment components for the probability of an animal being docile across the years. **a** GEBV of three sires with habituation (points), sensitization (square), and neutral (triangle) learning and behavioral plasticity. **b** GEBV of a sire (light blue line) and its daughter that have at least two CT records and have siblings in the same contemporary group. **c**–**e** Permanent environment impact (PE) of daughters of a single sire raised in same conditions (same contemporary group). EBV: probability of being docile at a genetic level; PE: impact of the permanent environment on the probability of being docile

A strong positive genetic correlation was observed between average CT and learning and behavioral plasticity (0.744), while a weak positive phenotypic correlation was observed between them (0.219). Figure 5a is a representation of nine bulls with more than 25 daughters with three learning and behavioral plasticity patterns: (i) habituation (positive slope), (ii) neutral (slope close to zero), and (iii) sensitization (negative slope). The Y-axis is the GEBV for the probability of the animal being docile. Figure 5b shows the additive genetic component for the learning and behavioral plasticity of one of the sires presented in Fig. 5a and a sample of its daughters (i.e., daughters with at least two CT records; average and maximum equal to 4 and 10 records, respectively). Figure 5c–e represents the impact of the permanent environment component on the probability of being docile for the daughters raised in similar conditions (i.e., panels (c), (d) and (e) represent three different CG).

### Genome-wide association for temperament across the years, average of cow temperament measured at weaning, and learning and behavioral plasticity

#### Longitudinal temperament

**Fig. 6:**
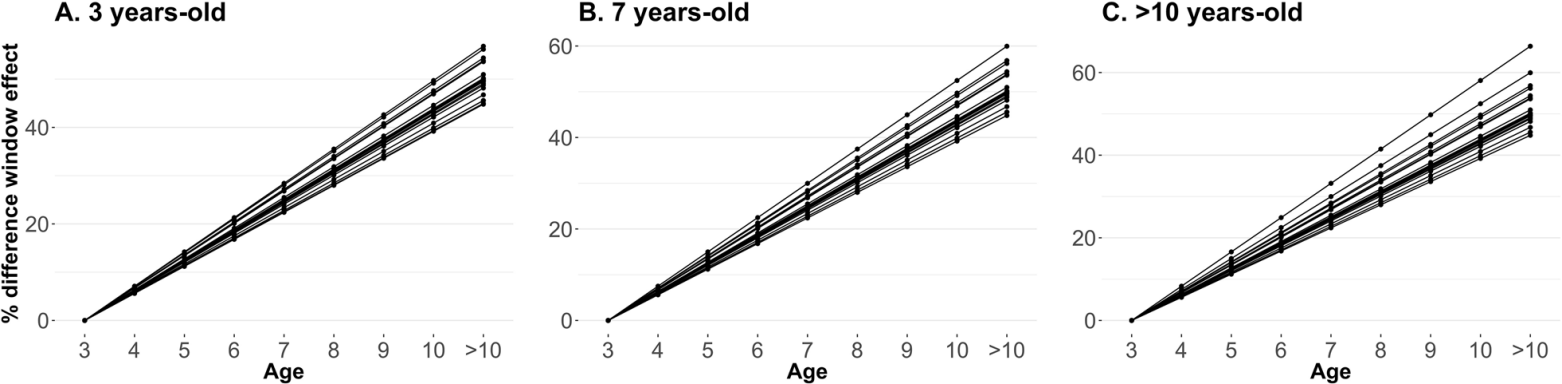
Percentage differences of SNP-window effect across the years for the top 20 genomic regions selected based on the effect for 3, 7, and > 10 years age groups. The liability effect was converted, in which increasing values mean becoming more docile. The SNP-window effect for age group 3-years-old was considered as the baseline for comparison

The intercept and slope’s SNP-window effect from RRM_CT were transformed to each age group’s SNP-window effect. The effect was used instead of the variance explained because the latter cannot be linearly transformed using the Legendre orthogonal polynomial coefficients. The top 20 genomic windows were selected for each age group based on the absolute effect. Top genomic regions overlapped a lot among age groups [from 90 to 100%; (see Additional file 1: Table S2)]. The Manhattan plot for each age group is presented in Additional file 2: Fig. S1). The percentage difference of the genomic window effect across the years is represented by the top regions for three age-groups (i.e., 3, 7, and > 10 years-old) in Fig. 6, in which the effect for the age group ‘3 years-old’ was chosen as the baseline (i.e., considered as 0).

**Fig. 7:**
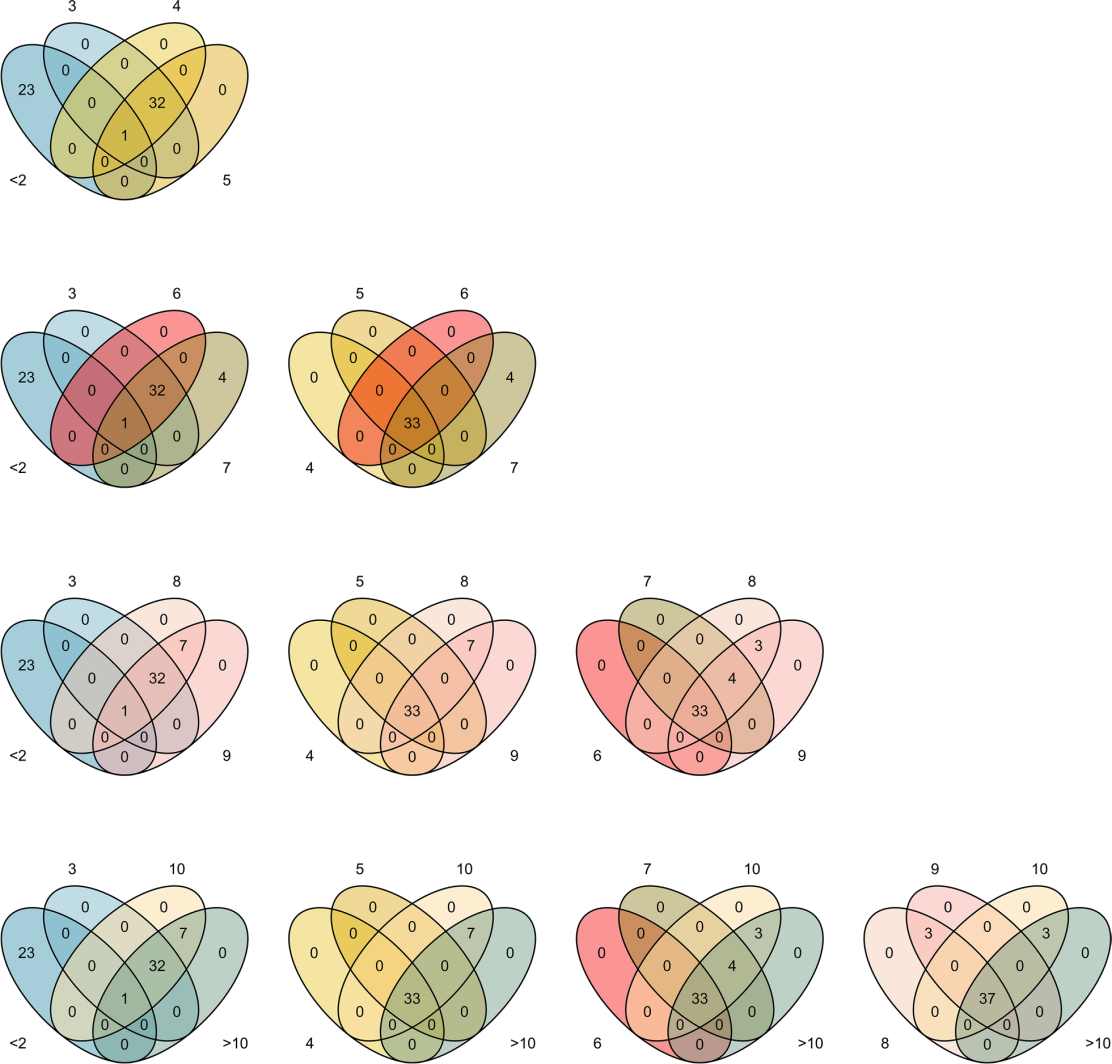
Venn diagram for the overlapping genes identified for the top 20 SNP-window based on absolute effect for the cow at weaning temperament and genes identified by Alvarenga et al. [5] for yearling temperament

Candidate genes surrounding the top 20 SNP-windows for each age group were retrieved. Thereafter, we identified the overlapping candidate genes among age groups (Fig. 7). One gene located on BTA10 i.e. *serine palmitoyl transferase long chain base subunit 2* gene (*SPTLC2*) overlapped between YT (< 2 years old) and CT (from 3 to > 10 years old). In addition, two paralogous genes also overlapped between YT and CT but were not captured by the Venn Diagram i.e. for the *U6* gene (*U6 spliceosomal RNA*), a paralogue being located on BTA8 for YT and another one on BTA10 for CT. Among CT age groups, 33 of the 43 genes identified overlapped among themselves (Fig. 7) and (see Additional file 1: Table S3). Seven of the 10 genes that did not overlap among the age groups were annotated, and they were located on BTA10 (e.g., *NREP*, *TRIP4*, *CSNK1G1, PCLAF*) and BTA18 [e.g., *BREH1*, *NUP90*, *CES5A*; (see Additional file 1: Table S3)].

#### Learning and behavioral plasticity

**Fig. 8:**
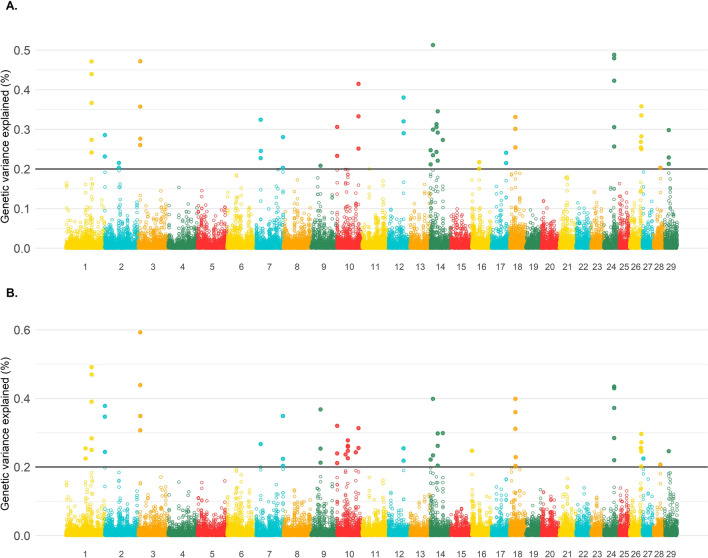
Manhattan plot for the average cow temperament measured at weaning (**a**) and learning and behavioral plasticity (**b**) components of a random regression model for cow temperament measured at weaning

Twenty-three and 26 non-overlapping SNP-windows explained more than 0.20% of the total additive genetic variance for the average CT and learning and behavioral plasticity, respectively. The maximum variance explained by the SNP-windows were 0.51 and 0.59% for the average CT and learning and behavioral plasticity, respectively. The Manhattan plots for both average CT and learning and behavioral plasticity are in Fig. 8. For the average CT, the top genomic regions are located on BTA1, 2, 3, 7, 9, 10, 12, 14, 16, 17, 18, 24, 26, 28 and 29, and those regions explained 7.46% of the total additive genetic variance. The genomic regions for the learning and behavioral plasticity are located on BTA1, 2, 3, 7, 9, 10, 14, 16, 18, 24, 26, 27, 28, and 29, explaining 8.10% of the total genetic variance. There were 18 genomic regions that overlapped between the average CT and learning and behavioral plasticity, leaving BTA12, 16 and 17 as unique regions for the average CT, and BTA16, 24, and 27 for the learning and behavioral plasticity.

Seventy-three annotated genes (63 protein coding) were located around the genomic regions for the average CT, and 98 annotated genes for the learning and behavioral plasticity (90 protein coding). A complete list of the genes is in Additional file 1: Table S4. Sixty-nine annotated genes were commonly identified for both average CT and learning and behavioral plasticity (61 protein coding genes), four genes were uniquely identified for the average CT (2 protein coding genes), and 29 genes were uniquely identified for the learning and behavioral plasticity (29 protein coding genes). Table 5 shows the most relevant candidate genes based on previous associations.

**Table 5:** Sample of the candidate genes identified for average cow temperament measured at weaning (average CT), learning and behavioral plasticity (LBP), and cow temperament across the years (CT)

Chr: Start–End bp	Gene	Gene name	Trait
1:80,873,608–81,097,089	*DGKG*	*Diacylglycerol kinase gamma*	LBP
10:36,050,714–36,074,743	*BAHD1*	*Bromo adjacent homology domain containing 1*	LBP
10:36,139,789–36,160,950	*CCDC32*	*Coiled-coil domain containing 32*	LBP
10:36,077,444–36,079,398	*CHST14*	*Carbohydrate sulfotransferase 14*	LBP
10:45,660,900–45,811,429	*CSNK1G1*	*Casein kinase 1 gamma 1*	LBP; CT 10 and > 10 years
10:2,213,062–2,245,525	*NREP*	*Neuronal regeneration related protein*	CT 8 and 9 years
10:88,689,279–88,784,551	*SPTLC2*	*Serine palmitoyltransferase long chain base subunit 2*	Average CT; LBP
10:45,556,247–45,639,746	*TRIP4*	*Thyroid hormone receptor interactor 4*	LBP; CT 10 and > 10 years
14:24,946,881–25,258,596	*TOX*	*Thymocyte selection associated high mobility group box*	Average CT
18:25,595,310–25,641,535	*ADGRG1*	*Adhesion G protein-coupled receptor G1*	LBP
18:24,682,781–24,710,853	*CES5A*	*Carboxylesterase 5A*	Average CT; LBP; CT from 7 to > 10 years

Considering the genes identified for both average CT and learning and behavioral plasticity altogether, four GO biological terms were statistically significant (P-value < 0.05), including cortisol metabolic process, steroid hormone biosynthetic process, aldosterone biosynthetic process, and cellular response to peptide hormone stimulus (see Additional file 1: Table S5). Other biologically relevant terms are glucocorticoid biosynthetic process (P-value < 0.10), dopamine receptor signaling pathway, negative regulation of receptor activity, and positive neural precursor cell proliferation.

Steroid 11-beta-monooxygenase activity and corticosterone 18-monooxygenase activity were the two GO molecular functions that were statistically significant [P-value < 0.05; (see Additional file 1: Table S5)]. No KEGG pathway terms were significant in this analysis, however, cholinergic synapse, melanogenic, and cortisol synthesis and secretion are three biologically relevant terms (see Additional file 1: Table S5).

The genomic regions with a variance that explained more than 0.20% for the average CT and learning and behavioral plasticity overlapped with 739 and 799 QTL-regions annotated for 78 and 88 traits in cattle, respectively (see Additional file 1: Table S6). Some examples of traits in which those QTL had previously been associated are bovine tuberculosis susceptibility, calf size, calving ease, clinical mastitis, conception rate, feet and leg conformation, ketosis, length of productive life, milking speed, twinning, and stillbirth.

### (Co)variance components of yearling and cow temperament measured at weaning

High genetic correlations were observed among all time-points for CT, which meets one of the assumptions to use a repeatability model. Posterior means and standard deviations for the variance components for CT in a repeatability model were obtained from 10,000 samples, which were a subset from a run with 200,000 iterations, 100,000 burn-in, and a thinning interval of 10. The posterior means and standard deviation for the heritability and repeatability estimates of CT were 0.44 (0.02) and 0.50 (0.01), respectively. The variance components are in Additional file 1: Table S7.

The genetic correlation between YT and CT was obtained using a bivariate model. The (co)variance components are in Table 6 and Additional file 1: Table S8. The heritability estimates in a two-trait model were similar to the estimates of a single-trait model, which were 0.42 for YT and 0.45 for CT (see Additional file 1: Table S8). The genetic and phenotypic correlation between YT and CT were 0.84 (0.01) and 0.37 (0.02), respectively.

**Table 6:** (Co)variance components for yearling and cow at weaning temperament

Components	Mean	SD
YT Heritability	0.42	0.01
CT Heritability	0.45	0.01
CT Repeatability	0.50	0.01
YT and CT genetic correlation	0.84	0.01
YT and CT phenotypic correlation	0.37	0.02

YT: yearling temperament; CT: cow temperament measured at weaning

## Discussion

### Phenotypic assessment of temperament over the years

Young calves, such as at weaning stage, usually show a fearful response to handling due to the novelty of being handled coupled with greater exposure to a variety of potential stressors including breaking the dam-calf bond, change of diet, and re-grouping with unfamiliar animals, as described by Enríquez et al. [44]. Furthermore, studies have shown that the average temperament score decreases as the frequency of handling or additional animal–human interaction increases, as long as the stockperson uses a calm approach to handling the animals [3, 5, 45, 46]. In other words, one would expect the average temperament to decrease (animals become more docile) as the age increases. On the one hand, experimentally-designed datasets are often associated with frequent human–animal interactions. On the other hand, in a seedstock/commercial setting, the handling frequency (i.e., human–animal interactions) decreases as the animals get older. With that, temperament scores taken on mature animals in this dataset were collected at or near the time calves were weaned which could lead to elevated stress responses while handling. Consequently, in practical scenarios an opposite behavioral pattern could be observed: the average temperament scores slightly increased as the animals aged (Fig. 2a), suggesting a sensitization across the years instead of habituation. In short, habituation can be defined as decreased responsiveness to a stimulus while sensitization is an increased responsiveness to a stimulus [9]. Habituation is often associated with adaptation [9].

We compared younger animals (YT, < 2 years-old) versus older than 2 years (CT, 3 years-old and older), in which differences can be attributed to factors related to the age and the recording event. First, 320 days is the minimum age when animals were recorded for temperament (YT), allowing the calves to acclimate to the challenging post-weaning environment [5]. Second, temperament at ages older than 2 years was recorded on cows around the weaning event. Cows differ in maternal behavior and in their response to weaning of their calf [47, 48], and separation from the calf is likely to trigger different motivations and physiological and behavioral responses during recording of CT compared to YT, although cows had typically been weaned a few weeks before recording of CT. Cows could also change their behavior to a flightier behavior (e.g., scores 2 or 3; Table 2) as a consequence of mothering instincts, in order to be more protective of their progenies.

The average cow at weaning temperament score (animals older than 2 years) increased slightly with age (Fig. 2a). The magnitude of changes of temperament scores between adjacent age groups was slightly greater between YT to CT (increased by 12%) than within CT (maximum increase between adjacent years equal to 2%), which also supports the argument that different lifetime events might evoke different motivational systems and capture context-specific behavioral responses. Furthermore, supporting these results, the phenotypic correlations between YT and CT were weakly positive [0.37; Tables 6 and (see Additional file 1: Table S8)], while strong phenotypic correlations were observed between CT scored in adjacent years and weaker correlations were estimated between larger age-group gaps (from 1.00 to 0.18; Fig. 4b).

Previous negative experiences can trigger subsequent fearful responses to handling due to memory acquisition [9, 16, 17, 49]. Although the number of human–animal interactions is expected to cumulatively increase across the animals’ lifetime, the frequency of interactions per year can decrease (e.g., until yearling the calves are intensively and routinely managed while older animals could be handled less than three times a year). An alternative hypothesis is that animals got, on average, flightier over time due to memorization of cumulative experiences. Such increased reactions to handling can impact the animal and handler welfare as well as impact the behavior of young replacement animals in the herd, because more temperamental dams also raise, on average, more temperamental progeny (Fig. 2b).

Another potential justification for younger animals to be less flighty than older animals is selection. Direct genetic selection based on the official genetic evaluation for temperament would result in a more docile younger population. Based on this theory, flightier animals would be censured on later-in-life temperament measurements. However, in practice, selection is not based on a single trait but rather on a selection index (group of traits) and thus, only animals with extremes breeding values for temperament might have been culled.

### Genetic and genomic assessment of temperament over the years

A strong positive genetic correlation was observed between YT and CT (0.84, Table 6) and between pairs of year-groups for CT (average equal to 0.98, Fig. 4). Although these correlations are strong, we observed a similar pattern of phenotypic correlations: stronger within age-groups of CT than between YT and CT. Furthermore, one out of the 24 genes identified for YT by Alvarenga et al. [5] overlapped with genes located within the top 20 genomic regions based on absolute effect for each age-group for CT (Fig. 8).

The overlapping *U6* paralog gene is a noncoding RNA. Alvarenga et al. [24] systematically reviewed genes that control behavioral indicators in mammalian livestock, and *U6* paralogous genes had been previously associated with three behavioral indicators in cattle and two indicators in pigs. Furthermore, a haplotype-based GWAS on yearling temperament in Angus cattle also identified *U6* as a candidate gene controlling yearling temperament [23].

The main overlapping gene between YT and CT, *SPTLC2,* is located on BTA10 and is associated with neurological diseases in humans [50] and re-learning mechanisms in rats [51]. The *SPTLC2* gene plays a role in the de novo synthesis of sphingolipids (i.e., responsible for signal transduction) by condensing L-serine and palmitoyl-coenzyme A into 3-keto sphinganine, which can be further converted into sphingoid bases [50]. Acid sphingomyelinase (ASM) is a key enzyme in sphingolipid metabolism, and decreased activity of ASM in the dorsal hippocampus has been associated with efficient re-learning in rats [51]. Interestingly, Huston et al. [51] also reported changes in the ASM enzymes as the rats aged.

Many genes identified for the top 20 SNP-windows based on the absolute value were common across age groups (Fig. 7) and (see Additional file 1: Table S3), which supports the strong genetic correlations observed between CT age groups. Although the genes controlling CT are similar, their effect can be time-dependent. Oliveira et al. [52] observed an increase and decrease in the magnitude of the SNP effects across time for milk yield and somatic cell score in dairy cattle, respectively. In our study, the magnitude of SNP-window effect increased with age for the top 20 genomic regions (Fig. 6). Furthermore, studies on mental disorders in humans have shown that genes become activated in response to life circumstances, consequently, initiating adverse behaviors [11]. In other words, we hypothesize that as the animal ages, behavioral-related genes are differentially regulated and/or their expression is altered. However, no conclusion can be drawn from the present study. Gene expression studies should be conducted on the genes identified to investigate this hypothesis.

In the same context, the genes that did not completely overlap with all age groups were identified later in life, and in general, they have been previously associated with age-related neural and mental disorders in humans and mice, such as memory and Alzheimer’s disease in older mice. The *carboxylesterase 5A* gene (*CES5A*) is located on BTA18, and it was identified as a candidate gene based on the top 20 SNP-regions for cows older than 7 years (see Additional file 1: Table S3). *CES5A* has been validated as a regulator of male mice fertility [53, 54]. As an expected role of carboxylesterase family genes, the CES5A protein can result in high levels of cholesterol and choline esterase [55], alterations of which impact cognitive impairment, especially on memory in aging rats [56–58].

The *thyroid hormone receptor interactor 4* (*TRIP4*), *casein kinase 1 gamma 1* (*CSNK1G1*), and *PCNA clamp associated factor* (*PCLAF*; also known as *PAF* and *PAF15*) genes are located on BTA10, and they were also identified as candidate genes controlling the expression of temperament later in life [animals older than 10 years; (see Additional file 1: Table S3)]. *TRIP4* gene has been identified as a candidate gene for Alzheimer’s disease susceptibility [59], and the *CSNK1G1* gene may be a cause of syndromic developmental delay and autism spectrum disorder [60]. Interestingly, a role for *TRIP4* gene has also been identified in behavioral maturation in honeybees, which comprises the labor-division among worker bees across their lifetime [61]. Behavioral maturation can be seen in the hierarchical emergence of activities of the work-bees over time, for example, from “*2–3 weeks of adult life, workers perform tasks inside the hive, such as nursing and food storage, and as they become older, they progress to tasks outside, including foraging for pollen and nectar*” [61].

The *neuronal regeneration related protein* gene (*NREP*; also known as *C5orf13*, *D4S114*, *P311*, *PRO1873*, *PTZ17*, or *SEZ17*) was identified as a candidate gene controlling behavioral responses at around 8 and 9 years-old (see Additional file 1: Table S3), and *NREP* plays an important role in the transforming growth factor beta (TGF-$\beta$) pathway [62]. In pigs, the *NREP* gene was indicated to improve meat production based on a gene expression study comparing Czech Large White pigs and wild boars [63]. Based on the same TGF-$\beta$ pathway, the *NREP* gene has been identified as a rare variant in about 20% of suicide patients, based on a whole-exome ultra-high throughput sequencing analysis in brain samples of human suicide victims [64].

### Genetic and genomic assessment of average temperament and learning and behavioral plasticity in cows

The Legendre orthogonal polynomial of first order was used to evaluate the average cow temperament measured at weaning and learning and behavioral plasticity, in which they are indicated by the intercept and slope of the random regression model, respectively. A similar approach was used in chickens, in which the authors showed that laying floor eggs and perching can be learned and there is a genetic component associated with it [65].

Learning and behavioral plasticity explains a low proportion of the total variation in cow temperament (3.33%). However, there is a genetic component associated with it, conveying that genetic and genomic selection could be applied to improve the magnitude of habituation over time, consequently, achieving the breeding goal of more docile animals. Moreover, learning and behavioral plasticity that leads to a favorable change in behavior (i.e., habituation rather than sensitization) may have a positive impact on animal welfare, both on farms that rely on frequent human–animal interaction, and high-tech farms. There is genetic variation in the learning and behavioral plasticity spectrum from habituation to sensitization among individuals, as shown in Fig. 5a. Both outcomes likely depend on well-developed cognitive abilities of learning, memory, and potentially individual human recognition, but differ in appraisal of threat. The top three sires in Fig. 5a (circle points) produced progeny that have a desirable expression of learning and behavioral plasticity because they habituate to handling. The contrasting sires (square points) produce progeny with an undesirable expression of learning and behavioral plasticity manifested by sensitization.

A wide range (from 0.02 to 0.99) of heritability estimates have been reported for cognitive performance across many species, including humans, chimpanzee, rhesus macaque, domestic pig, rat, mouse, zebra finch, honey bees, guppy, and jungle fowl [18–22, 61]. In this study, a low heritability was observed for the learning and behavioral plasticity component (0.02 $\pm$ 0.002; Table 4). Strategies such as the use of genomic information [66, 67] and quantification of specific cognitive attributes (e.g., appropriate matching of threat appraisal with actual risk of harm) might assist and advance genetic improvement for learning and behavioral plasticity. Furthermore, in this study most cows had only one record, which could compromise the estimation of the slope in the RRM, consequently, rendering lower heritability for the learning and behavioral plasticity. However, we have done an additional analysis including only animals with more than two records and no major differences were observed in the estimation of the slope heritability [learning and behavioral plasticity; (see Additional file 1: Tables S10 and S11)].

The permanent environment effect explains the larger part of the variance of learning and behavioral plasticity, indicating that the experiences that the animal had throughout their life are a greater determinant of learning and behavioral plasticity between individuals. As discussed before, the environment may also induce changes in the expression (and importance) of certain genomic regions. Different rearing conditions can lead to distinct experiences, as seen in Fig. 5c–e. However, even within the same rearing conditions, animals can have individual experiences, and, consequently, different impacts on their behavioral response (shown in the animals in Fig. 5c–e).

A moderate-to-high genetic correlation was observed between the average CT and learning and behavioral plasticity. Accordingly, many of the annotated candidate genes (68%) identified overlapped between both components. The learning and behavioral plasticity had more genes uniquely identified [29 genes: e.g., (BTA1) *DGKG*; (BTA10) *BAHD1, CCDC32, CHST14, CSNK1G1, FUT8, TRIP4;* (BTA16) *KISS2, PIK3C2;* (BTA18) *ADGR1, ADGR3*, *ADGR5;* (BTA24) *IMPA2*] compared to the average CT (four genes: *TOX, SMUD3, U6, 5S_rRNA*).

For the average CT, two of the four uniquely identified genes were associated with mental disorders in humans and behavioral traits in livestock species. The *thymocyte selection associated high mobility group box* gene (*TOX*) has an important role in regulating neural stem cell proliferation in neural tissues [68], and differential expression was seen between patients with schizophrenia and control patients (accuracy of 86% [69]). Another gene previously described, *U6*, is a paralog gene associated with adrenaline levels, feeding behavior, maternal behavioral, suckling reflex, and temperament (including Angus yearling temperament) in cattle and pigs [5, 23, 24].

The cognitive-related candidate genes were previously associated with development delay, impaired learning and behavioral plasticity, and other mental and neuronal disorders in humans. The *diacylglycerol kinase gamma* gene (*DGKG*) was reported to be negatively regulated by the triiodothyronine hormone in mice inactivated for astrocyte-specific *Dio2*, which resulted in mood and behavioral disorders, such as anxiety-depressive behavior [70]. Similarly, *BAHD1* has also been suggested to regulate brain cells and, consequently, to be linked to anxiety-like behavior [71]. Comparing *BAHD1-*knockout and control mouse groups, the authors identified many differentially expressed genes in the brain suggesting a role for *BAHD1* in transcriptional regulation in neural tissue [71]. Interestingly, 52% of the annotated cognitive-related genes are from the same family of some differentially expressed genes found in *BAHD1-*knockout mice, including the *adhesion G protein-coupled receptor G1* (*ADGRG1*), *coiled-coil domain-containing* (CCDC family; including *CCDC102A*), *carbohydrate sulfotransferase 11* (CHST family), *casein kinase 1 gamma* (CSNK1G family), and *thyroid hormone receptor interactor* (TRIP family) genes.

Three adhesion G protein-coupled receptor G (ADGRG) family genes had been identified as cognitive-specific candidates (see Additional file 1: Table S4), which have crucial neurodevelopment functions [72]. Furthermore, *ADGRG1* knockdown mice were associated with depression-like behavior, executive dysfunction, and poor response to neurological treatment (e.g., antidepressant [73]). The *carbohydrate sulfotransferase 14* (*CHST14*), *casein kinase 1 gamma* (*CSNK1G1*), and *thyroid hormone receptor interactor* (*TRIP4*) gene families were previously associated with cognitive performance in mice and honeybees [60, 61, 74]. The *CSNK1G1* and *TRIP4* gene functions and associations were described above as they were implicated in CT of cows of more than 10 years of age.

Knockdown mice for the *CHST14* gene were linked to spatial learning and memory impairment with the gene action located in the hippocampus [74]. Furthermore, the *CHST14* and *CCDC32* genes have been identified in a genome-wide-gene-by-environment interaction as a carrier for high risk of suicide, primarily driven by post-traumatic stress in woman [75]. The majority of the genes identified in this study sustain their function on behavior and cognitive performance across adult life. However, the *CHST14* and *CCDC32* genes support the hypothesis that genomic regions can change their effect and/or impact over time due to specific experiences, as shown in Fig. 8. Further validation studies on this longitudinal behavior should be carried out.

In general, the adrenal glands release two major hormones observed during stress responses: cortisol or corticosterone and aldosterone. Physiological stress-driven events activate the hypothalamic–pituitary–adrenal (HPA) axis signaling the adrenal glands. Cortisol is the primary steroid hormone to cope with stressors in some mammals [76]. However, concomitantly with the HPA, other systems are also activated, for example, the sympathetic-adrenomedullary system leading to increased secretion of aldosterone [77]. Excessive stressors can impair many essential mechanisms, including personality development and behavior [76]. For instance, chronic excess of cortisol and aldosterone concentrations can lead to cognitive disorders, such as suppressed memory retrieval [77, 78]. Pathways and mediators for these steroid hormones, cortisol and aldosterone, were enriched in this study, such as cortisol metabolic, aldosterone and glucocorticoid biosynthetic, and cellular response to peptide hormone stimulus (see Additional file 1: Table S5).

### Implications and next steps

The covariance structure among CT age groups was obtained from RRM fitting Legendre orthogonal polynomial of first order, in which we assumed homogeneous residual variances across age groups. This assumption was made due to computational and software limitations, but future studies should model the phenotypic variation across the years considering heterogeneous residual variance. Furthermore, the model used considered a constant permanent environment impact on the phenotype, which, for behavioral traits, may not be true. Throughout the animals’ life they could have a negative followed by a positive experience, resulting in a null permanent environmental effect because these events cancelled each other out. Therefore, it would be advantageous to evaluate correlations among ages fitting a cumulative permanent environment model for a longitudinal behavioral study [79]. Furthermore, given that either phenotypic or genetic selection might be applied based on docility in combination with other economically important traits early in life, animals with extreme temperament might be prematurely culled and therefore, might have less phenotypic records on later age-groups (censored data). Therefore, models considering the censured nature of the data should be evaluated to account for potential pre-selection bias.

The first-order Legendre orthogonal polynomial provided the opportunity to evaluate learning and behavioral plasticity in cows over the years, which can be an indicator trait to improve the animal and handler-welfare in the long-term, as well as selection of animals that would habituate to the emerging technology on farms assuming that habituation to handling predicts habituation to other management related stimuli. Another reason for the first-order Legendre orthogonal polynomial fitted was data limitation (i.e., average repeated records per animal equal to two). Therefore, continuing to record docility scores at yearling and on older animals will be essential. Other Legendre polynomial orders could be tested in future analyses (when more records per cow are available) to identify the optimal behavioral pattern across a cow’s lifetime.

Learning and behavioral plasticity was defined by the data available; in other words, no new data recording other than already being collected by farmers was used. However, low heritability was observed providing opportunities for the identification of other optimal indicators with higher heritability within the scope of easy implementation. Furthermore, as a consequence of the high genetic correlation between overall temperament (i.e., intercept) and learning and behavioral plasticity (i.e., slope; Table 4), the current selection strategy on temperament results in indirect selection for learning and behavioral plasticity. The current genetic selection for YT would also indirectly improve the docility of cows at different ages. In addition, the inclusion of additional temperament records collected later in life into the YT genomic evaluations might increase the accuracy of the breeding values.

This is the first study evaluating the changes of genomic-region effect on cattle temperament across the animal’s life. There is variation in genomic region effect over the years for temperament (Fig. 6), and genes associated with aging-related-diseases were located in those regions. This information is key for understanding behavioral changes in a cow’s life, as well as providing opportunities to identify environmental stimulators associated with those alterations. Further longitudinal gene expression studies are key to understanding their effect on temperament, the epigenomic regions involved, and to pinpoint environmental events regulating their expression.

## Conclusions

Temperament is moderately-to-highly heritable throughout the animals’ life, suggesting that an improvement in the herd-temperament score would be observed as a consequence of genetic or genomic selection. There is also a genetic component associated with learning and behavioral plasticity as expressed by habituation, although heritability is very low. Yearling and cow at weaning temperament, and among age groups for cow at weaning temperament are highly genetically correlated. We identified several potential candidate genes associated with learning and behavioral plasticity although they explain a small proportion of the genetic variance. In addition, we suggest that the impact of the genes on behavior might change over time due to gene-environment-interactions. The present study resulted in a better understanding of the genetic and genomic mechanisms underlying temperament across the life of beef cows. Moreover, we presented an indicator of learning and behavioral plasticity that offers opportunities to improve the long-term animal welfare. Future farms will need cattle that can habituate readily in spite of receiving less human contact than currently, and therefore, this area of research needs to be further explored.

## Supplementary Information

**Additional file 1: Table S1**. Variance components over time for cow at weaning temperament using a random regression model. **Table S2.** Number of overlapping genomic regions among the top 20 windows with the highest module effect for cow at weaning temperament. **Table S3**. Genes within the top genomic regions for cow at weaning temperament across the age groups. **Table S4**. Genes identified for the average cow at weaning temperament and learning and behavioral plasticity for cow at weaning temperament. **Table S5.** Functional annotation for genes controlling average of cow at weaning temperament and learning and behavioral plasticity. **Table S6.** Quantitative trait loci that overlap with the genomic regions for the average of cow at weaning temperament (inter) and learning and behavioral plasticity (slope). **Table S7**. Variance components for cow at weaning temperament using a repeatability model. **Table S8**. (Co)variance components for yearling and cow at weaning temperament using a two-trait model. **Table S9.** (Co)variance components for yearling and cow at weaning temperament as a single-trait repeatability model. **Table S10.** (Co)variance components for average cow at weaning temperament (AverCT) and learning and behavioral plasticity (LBP) of a dataset containing animals with more than two records. **Table S11.** Variance components over time for cow at weaning temperament using a random regression model and data of animals with at least two records.

**Additional file 2: Figure S1.** Manhattan plot for each age group of cows at weaning temperament (CT) considering the absolute effect of SNP-window.

## Data Availability

The data supporting the results of this article are included within the article and in its supplementary files. The raw data cannot be made available, as it is property of the American Angus cattle producers, and this information is commercially sensitive.
