# Supplementary material for: Unraveling the phenotypic and genomic background of behavioral plasticity and temperament in North American Angus cattle
Source: Genet Sel Evol. 2023 Jan 19;55:3. doi: 10.1186/s12711-023-00777-3 (PMC9850537; doi:10.1186/s12711-023-00777-3)
Supplement: Supplementary file 1 — Additional file 1: Table S1. Variance components over time for cow at weaning temperament using a random regression model. Table S2. Number of overlapping genomic regions among the top 20 windows with the highest module effect for cow at weaning temperament. Table S3. Genes within the top genomic regions for cow at weaning temperament across the age groups. Table S4. Genes identified for the average cow at weaning temperament and learning and behavioral plasticity for cow at weaning temperament. Table S5. Functional annotation for genes controlling average of cow at weaning temperament and learning and behavioral plasticity. Table S6. Quantitative trait loci that overlap with the genomic regions for the average of cow at weaning temperament (inter) and learning and behavioral plasticity (slope). Table S7. Variance components for cow at weaning temperament using a repeatability model. Table S8. (Co)variance components for yearling and cow at weaning temperament using a two-trait model. Table S9. (Co)variance components for yearling and cow at weaning temperament as a single-trait repeatability model. Table S10. (Co)variance components for average cow at weaning temperament (AverCT) and learning and behavioral plasticity (LBP) of a dataset containing animals with more than two records. Table S11. Variance components over time for cow at weaning temperament using a random regression model and data of animals with at least two records. [file 12711_2023_777_MOESM1_ESM.docx]

**Additional Tables**

**Table S1** Variance components for cow at weaning temperament over time using a random regression model in North American Angus cattle.

|  | $\sigma_{CG}^{2}$ | | $\sigma_{U}^{2}$ | | $\sigma_{PE}^{2}$ | | $\sigma_{e}^{2}$ | | $h^{2}$ | | Rep | |
| --- | --- | --- | --- | --- | --- | --- | --- | --- | --- | --- | --- | --- |
| **Age** | **Mean** | **SD** | **Mean** | **SD** | **Mean** | **SD** | **Mean** | **SD** | **Mean** | **SD** | **Mean** | **SD** |
| 3 | 0.016 | 0.002 | 0.125 | 0.006 | 0.061 | 0.005 | 0.129 | 0.008 | 0.379 | 0.018 | 0.563 | 0.012 |
| 4 | 0.011 | 0.001 | 0.140 | 0.006 | 0.058 | 0.004 | 0.129 | 0.008 | 0.414 | 0.016 | 0.586 | 0.012 |
| 5 | 0.007 | 0.001 | 0.157 | 0.006 | 0.059 | 0.005 | 0.129 | 0.008 | 0.446 | 0.016 | 0.613 | 0.012 |
| 6 | 0.004 | 0.001 | 0.176 | 0.007 | 0.063 | 0.005 | 0.129 | 0.008 | 0.473 | 0.018 | 0.643 | 0.012 |
| 7 | 0.002 | 0.001 | 0.198 | 0.009 | 0.072 | 0.007 | 0.129 | 0.008 | 0.494 | 0.020 | 0.673 | 0.012 |
| 8 | 0.001 | 0.001 | 0.222 | 0.012 | 0.084 | 0.009 | 0.129 | 0.008 | 0.509 | 0.023 | 0.702 | 0.012 |
| 9 | 0.001 | 0.001 | 0.249 | 0.015 | 0.100 | 0.011 | 0.129 | 0.008 | 0.519 | 0.026 | 0.729 | 0.012 |
| 10 | 0.002 | 0.001 | 0.278 | 0.019 | 0.120 | 0.014 | 0.129 | 0.008 | 0.525 | 0.029 | 0.753 | 0.012 |
| >10 | 0.003 | 0.002 | 0.309 | 0.022 | 0.144 | 0.018 | 0.129 | 0.008 | 0.528 | 0.032 | 0.774 | 0.011 |

$\sigma_{CG}^{2}$: contemporary group variance; $\sigma_{U}^{2}$: additive genetic variance; $\sigma_{PE}^{2}$: permanent environment variance; $\sigma_{e}^{2}$: residual variance; $h^{2}$*:* heritability; Rep: repeatability; SD: standard deviation.

**Table S2** Number of overlapped genomic regions among the top 20 windows with the highest module effect for cow at weaning temperament in North American Angus cattle.

|  | Age-group | | | | | | | |
| --- | --- | --- | --- | --- | --- | --- | --- | --- |
| Age-group | **>3** | **4** | **5** | **6** | **7** | **8** | **9** | **10** |
| >3 |  |  |  |  |  |  |  |  |
| 4 | 20 |  |  |  |  |  |  |  |
| 5 | 20 | 20 |  |  |  |  |  |  |
| 6 | 20 | 20 | 20 |  |  |  |  |  |
| 7 | 19 | 19 | 19 | 19 |  |  |  |  |
| 8 | 18 | 18 | 18 | 18 | 19 |  |  |  |
| 9 | 18 | 18 | 18 | 18 | 19 | 20 |  |  |
| 10 | 18 | 18 | 18 | 18 | 19 | 19 | 19 |  |
| >10 | 18 | 18 | 18 | 18 | 19 | 19 | 19 | 20 |

**Table S3** Genes within the top genomic regions for cow at weaning temperament across the age groups.

| Commonly identified genes among age-groups for cow at weaning temperament | | | |
| --- | --- | --- | --- |
| From 3 to 10+ | **From 7 to 10+** | **8 and 9** | **10 and 10+** |
| (BTA10)  *SOTLC2, ALKBH1, SLIRP, SNW1, U6, ADCK1*  (BTA14)  *GSDMC*  (BTA17)  ENSBTAG00000052755  (BTA18)  *U6*  (BTA24)  *GNAL, MPPE1, IMPA2, CIDEA, TUBB6, AFG3L2, PRELID3A, SPIRE1,*  ENSBTAG0000004692,  *CEP76, PSMG2, PTPN2, SEH1L, CEP192, LDLRAD4*  (BTA 26)  *OAT, NKX1-2, LHPP, ADAM12, C26H10orf90, MGMT,*  ENSBTAG0000005493,  *EBF3*  (BTA29)  *ANO5* | **(BTA18)**  *BREH1, NUP90, CES5A,*  ENSBTAG00000053753 | **(BTA10)**  *NREP,* ENSBTAG00000048541, ENSBTAG00000052475 | **(BTA10)**  *TRIP4, CSNK1G1, PCLAF* |

**Table S4** Genes identified for the average cow at weaning temperament and learning and behavioral plasticity for cow at weaning temperament in North American Angus cattle.

|  | Commonly identified | Uniquely identified | |
| --- | --- | --- | --- |
| Chr | **Average CT (intercept) and Learning/Behavioral Plasticity** | **Average CG (intercept)** | **Learning/Behavioral Plasticity** |
| BTA1 | *U6* |  | *DGKG* |
| BTA2 | ENSBTAG00000052889,  *CYFIP1, NIPA1, NIPA2* |  |  |
| BTA3 | ENSBTAG00000010458,  ENSBTAG00000015741, ENSBTAG00000046231, ENSBTAG00000051438, ENSBTAG00000054271, ENSBTAG00000048768, *CD244, CD48, CD84, COPA, LY9, NCSTN, PEX19, SLAMF1, SLAMF6, SLAMF7, U6, VANGL2* |  |  |
| BTA7 | ENSBTAG00000048456, ENSBTAG00000050565, ENSBTAG00000052694, ENSBTAG00000053476, ENSBTAG00000054046, ENSBTAG00000054165, *CAMK4, CHAF1A, CREB3L3, EBI3, FSD1, HDGFL2, MAP2K2, MPND, PLIN4, PLIN5, SH3GL1, SHD, SIRT6, STAP2, TMIGD2, UBXN6, YJU2* |  |  |
| BTA9 | ENSBTAG00000050641, *7SK, FYN, MFSD4B, REV3L, SLC16A10, TRAF3IP2* |  |  |
| BTA10 | ENSBTAG00000049115, ENSBTAG00000050601, ENSBTAG00000051430, *ALKBH1, SPTLC2* |  | ENSBTAG00000045360, ENSBTAG00000048559, ENSBTAG00000049725, *BAHD1, CCDC32, CHST14, CSNK1G1, FUT8, KNL1, PCLAF, RAD51, RMDN3, RPUSD2, TRIP4, ZNF609* |
| BTA14 | ENSBTAG00000004596, ENSBTAG00000037824, ENSBTAG00000049400, ENSBTAG00000052291, ENSBTAG00000054117, ENSBTAG00000054483, ENSBTAG00000036942, *ARMC1, CSMD3, CYP11B1, CYRIB, GLI4, GML, GPIHBP1, LY6E, LY6H, LY6K, MTFR1, PDE7A* | *TOX* |  |
| BTA16 |  | *SMYD3, U6* | ENSBTAG00000054185, *ETNK2, GOLT1A, KISS1, PIK3C2B, PLEKHA6, PPP1R15B, REN, SOX13* |
| BTA18 | ENSBTAG00000053753, *BREH1, CES5A, GNAO1* |  | *ADGRG1, ADGRG3, ADGRG5, CCDC102A* |
| BTA24 | *GNAL, MPPE1* | ENSBTAG00000028631 | *AFG3L2, CIDEA, IMPA2, TUBB6* |
| BTA26 | *DOCK1, MGMT* |  |  |
| BTA28 | ENSBTAG00000052269, *ANAPC16, ASCC1, SPOCK2, U6* |  |  |
| BTA29 | *TENM4* |  |  |

**Table S5** Functional annotation for genes controlling average of cow at weaning temperament and learning and behavioral plasticity.

| Category | Term | Count | Genes | FDR |
| --- | --- | --- | --- | --- |
| GOTERM_BP | GO:0034650~Cortisol metabolic process | 3 | ENSBTAG00000004596, ENSBTAG00000037824, *CYP11B1* | 0.023559 |
| GOTERM_BP | GO:0006700~C21-steroid hormone biosynthetic process | 3 | ENSBTAG00000004596, ENSBTAG00000037824, *CYP11B1* | 0.023559 |
| GOTERM_BP | GO:0032342~Aldosterone biosynthetic process | 3 | ENSBTAG00000004596, ENSBTAG00000037824, *CYP11B1* | 0.023559 |
| GOTERM_BP | GO:0071375~cellular response to peptide hormone stimulus | 3 | ENSBTAG00000004596, ENSBTAG00000037824, *CYP11B1* | 0.029346 |
| GOTERM_BP | GO:0006704~glucocorticoid biosynthetic process | 3 | ENSBTAG00000004596, ENSBTAG00000037824, *CYP11B1* | 0.065054 |
| GOTERM_BP | GO:0008203~cholesterol metabolic process | 3 | ENSBTAG00000004596, ENSBTAG00000037824, *CYP11B1* | 1 |
| GOTERM_BP | GO:0006307~DNA dealkylation involved in DNA repair | 2 | *ALKBH1*, *MGMT* | 1 |
| GOTERM_BP | GO:0010890~positive regulation of sequestering of triglyceride | 2 | *CIDEA*, *PLIN5* | 1 |
| GOTERM_BP | GO:0015693~magnesium ion transport | 2 | *NIPA1, NIPA2* | 1 |
| GOTERM_BP | GO:0007212~dopamine receptor signaling pathway | 2 | *NCSTN, GNAO1* | 1 |
| GOTERM_BP | GO:0050773~regulation of dendrite development | 2 | *CSMD3, DGKG* | 1 |
| GOTERM_BP | GO:2000272~negative regulation of receptor activity | 2 | *LY6H, RAD51* | 1 |
| GOTERM_BP | GO:0019985~translesion synthesis | 2 | *REV3L, PCLAF* | 1 |
| GOTERM_BP | GO:0010569~regulation of double-strand break repair via homologous recombination | 2 | *SIRT6, RAD51* | 1 |
| GOTERM_BP | GO:0001779~natural killer cell differentiation | 2 | *SLAMF1, TOX* | 1 |
| GOTERM_BP | GO:2000179~positive regulation of neural precursor cell proliferation | 2 | *TOX, ADGRG1* | 1 |
| GOTERM_BP | GO:0006446~regulation of translational initiation | 2 | ENSBTAG00000046231, *ALKBH1* | 1 |
| GOTERM_BP | GO:0007189~adenylate cyclase-activating G-protein coupled receptor signaling pathway | 3 | *ADGRG1, ADGRG5, ADGRG3* | 1 |
| GOTERM_MF | GO:0004507~steroid 11-beta-monooxygenase activity | 3 | ENSBTAG00000004596, ENSBTAG00000037824, *CYP11B1* | 0.005501 |
| GOTERM_MF | GO:0047783~corticosterone 18-monooxygenase activity | 3 | ENSBTAG00000004596, ENSBTAG00000037824, *CYP11B1* | 0.005501 |
| GOTERM_MF | GO:0035473~lipase binding | 2 | *GPIHBP1, PLIN5* | 0.937974 |
| GOTERM_MF | GO:0001664~G-protein coupled receptor binding | 3 | *FYN, GNAL, GNAO1* | 0.937974 |
| GOTERM_MF | GO:0030550~acetylcholine receptor inhibitor activity | 2 | *LY6E, LY6H* | 0.937974 |
| GOTERM_MF | GO:0042802~identical protein binding | 10 | *SLAMF1, FYN, CD84, SOX13, CHAF1A, FSD1, PLIN5, SH3GL1, SLAMF7, RAD51* | 0.968041 |
| GOTERM_MF | GO:0070851~growth factor receptor binding | 2 | *FYN, NCSTN* | 0.968041 |
| GOTERM_MF | GO:0004771~sterol esterase activity | 2 | *BREH1, CES5A* | 0.968041 |
| GOTERM_MF | GO:0015095~magnesium ion transmembrane transporter activity | 2 | *NIPA1, NIPA2* | 1 |
| GOTERM_MF | GO:0031683~G-protein beta/gamma-subunit complex binding | 2 | *GNAL, GNAO1* | 1 |
| GOTERM_MF | GO:0052689~carboxylic ester hydrolase activity | 2 | *BREH1, CES5A* | 1 |
| GOTERM_MF | GO:0044389~ubiquitin-like protein ligase binding | 2 | ENSBTAG00000049115, *TRIP4* | 1 |
| GOTERM_MF | GO:0038023~signaling receptor activity | 3 | *SLAMF1, CD244, CD48* | 1 |
| KEGG | bta04725:Cholinergic synapse | 4 | *CAMK4, FYN, CREB3L3, GNAO1* | 1 |
| KEGG | bta04650:Natural killer cell mediated cytotoxicity | 4 | *FYN, CD244, CD48, MAP2K2* | 1 |
| KEGG | bta04934:Cushing syndrome | 4 | *CREB3L3, MAP2K2,* ENSBTAG00000037824, *CYP11B1* | 1 |
| KEGG | bta04927:Cortisol synthesis and secretion | 3 | *CREB3L3*, ENSBTAG00000037824,  *CYP11B1* | 1 |
| KEGG | bta04070:Phosphatidylinositol signaling system | 3 | *PIK3C2B, DGKG, IMPA2* | 1 |
| KEGG | bta04916:Melanogenesis | 3 | *CREB3L3, MAP2K2, GNAO1* | 1 |

**Table S6** Quantitative trait loci overlapping with the genomic regions for the average of cow at weaning temperament (intercept) and learning and behavioral plasticity (slope).

| Trait | Chr | Coefficient | | Trait | Chr | Coefficient | | Trait | Chr | Coefficient | |
| --- | --- | --- | --- | --- | --- | --- | --- | --- | --- | --- | --- |
|  |  | **Inter** | **Slope** |  |  | **Inter** | **Slope** |  |  | **Inter** | **Slope** |
| Age at puberty | 14 | 3 | 1 | **Maturity rate** | 18 | 1 | 1 | **PTA type** | 16 | 0 | 3 |
|  | 1 | 0 | 1 | **Milk C14 index** | 14 | 11 | 11 |  | 17 | 1 | 0 |
| Average daily gain | 14 | 1 | 0 | **Milk C16 index** | 14 | 26 | 26 | **Rear leg placement - rear view** | 16 | 0 | 2 |
| Body capacity | 9 | 1 | 1 | **Milk C18 index** | 14 | 8 | 8 |  | 17 | 1 | 0 |
| Body depth | 16 | 0 | 3 | **Milk calcium content** | 14 | 3 | 3 |  | 14 | 1 | 1 |
|  | 9 | 1 | 1 | **Milk caprylic acid content** | 14 | 3 | 3 |  | 17 | 1 | 0 |
| Body weight (birth) | 14 | 6 | 1 | **Milk casein percentage** | 14 | 1 | 1 |  | 3 | 1 | 1 |
| Bovine tuberculosis susceptibility | 10 | 0 | 1 | **Milk cholesterol content** | 14 | 2 | 2 | **Shear force** | 10 | 0 | 1 |
|  | 12 | 1 | 1 | **Milk conjugated linoleic acid content** | 14 | 8 | 8 |  | 16 | 0 | 1 |
|  | 14 | 2 | 2 | **Milk fat percentage** | 14 | 120 | 120 |  | 18 | 0 | 1 |
|  | 29 | 3 | 3 |  | 3 | 1 | 1 |  | 7 | 5 | 5 |
| Calf size | 9 | 1 | 1 | **Milk fat yield** | 14 | 46 | 46 |  | 27 | 0 | 1 |
| Calving ease | 10 | 0 | 1 |  | 3 | 1 | 1 | **Somatic cell count** | 26 | 1 | 1 |
|  | 16 | 0 | 1 |  | 9 | 1 | 1 |  | 10 | 0 | 1 |
|  | 3 | 1 | 1 | **Milk fat-to-protein ratio** | 14 | 4 | 4 |  | 14 | 1 | 1 |
| Calving ease (maternal) | 16 | 0 | 2 | **Milk glycerophosphocholine to phosphocholine ratio** | 9 | 1 | 1 | **Stillbirth** | 10 | 0 | 1 |
|  | 3 | 1 | 1 | **Milk glycosylated kappa-casein percentage** | 10 | 1 | 1 |  | 16 | 0 | 2 |
| Carcass weight | 10 | 0 | 3 |  | 26 | 8 | 8 |  | 3 | 1 | 1 |
|  | 14 | 3 | 0 | **Milk iron content** | 14 | 2 | 2 | **Teat placement - front** | 14 | 1 | 1 |
|  | 3 | 1 | 1 | **Milk kappa-casein percentage** | 26 | 9 | 9 |  | 16 | 0 | 2 |
| Clinical mastitis | 14 | 1 | 1 | **Milk linoleic acid content**  **Milk linoleic acid content** | 14 | 29 | 29 | **Tenderness score** | 14 | 2 | 2 |
| Conception rate | 18 | 0 | 1 |  | 16 | 1 | 0 |  | 18 | 53 | 53 |
|  | 3 | 1 | 1 | **Milk linolenic acid content** | 14 | 20 | 20 |  | 9 | 1 | 1 |
|  | 9 | 1 | 1 | **Milk myristic acid content** | 14 | 12 | 12 | **Total bilirubin level** | 14 | 1 | 1 |
| Connective tissue amount | 14 | 2 | 2 | **Milk oleic acid content** | 14 | 34 | 34 | **Twinning** | 18 | 2 | 2 |
|  | 18 | 5 | 5 | **Milk palmitic acid content** | 14 | 35 | 35 | **Udder attachment** | 16 | 0 | 3 |
| Dairy capacity composite index | 14 | 1 | 1 |  | 27 | 0 | 1 |  | 17 | 1 | 0 |
| Dairy form | 10 | 0 | 1 | **Milk palmitoleic acid content** | 14 | 36 | 36 | **Udder cleft** | 10 | 0 | 1 |
|  | 3 | 1 | 1 | **Milk pentadecylic acid content** | 14 | 18 | 18 |  | 16 | 0 | 2 |
| Daughter pregnancy rate | 10 | 0 | 1 | **Milk phosphorus content** | 14 | 1 | 1 |  | 3 | 1 | 1 |
|  | 3 | 1 | 1 | **Milk protein percentage** | 10 | 0 | 4 | **Udder composite index** | 14 | 2 | 2 |
| Direct bilirubin level | 14 | 1 | 1 |  | 14 | 54 | 54 | **Udder depth** | 16 | 0 | 3 |
| Dry matter intake | 14 | 1 | 1 |  | 3 | 1 | 1 |  | 17 | 1 | 0 |
| Feet and leg conformation | 16 | 0 | 2 | **Milk protein yield** | 14 | 17 | 17 |  | 27 | 0 | 1 |
|  | 17 | 1 | 0 |  | 26 | 1 | 1 | **Udder height** | 10 | 0 | 1 |
| Foot angle | 16 | 0 | 2 |  | 3 | 2 | 2 |  | 16 | 0 | 3 |
|  | 17 | 1 | 0 |  | 9 | 1 | 1 |  | 17 | 1 | 0 |
|  | 3 | 1 | 1 | **Milk riboflavin content** | 14 | 10 | 10 | **Body weight gain** | 10 | 0 | 1 |
| Insulin-like growth factor 1 level | 14 | 2 | 0 | **Milk tridecylic acid content** | 7 | 1 | 1 | **Kidney, pelvic, and heart fat percentage** | 27 | 0 | 1 |
| Interval to first estrus after calving | 14 | 2 | 0 | **Milk unglycosylated kappa-casein percentage** | 12 | 5 | 5 | **Milk butyric acid content** | 16 | 0 | 3 |
| Ketosis | 14 | 8 | 8 | **Milk yield** | 10 | 2 | 2 | **Milk capric acid content** | 10 | 0 | 1 |
| Lactation persistency | 10 | 2 | 2 |  | 14 | 49 | 49 |  | 10 | 0 | 1 |
| LDL cholesterol level | 10 | 1 | 1 |  | 16 | 0 | 2 | **Milk medium-chain fatty acid content** | 10 | 0 | 1 |
| Length of productive life | 10 | 0 | 1 |  | 3 | 1 | 1 | **Rump width** | 16 | 0 | 3 |
|  | 3 | 1 | 1 |  | 9 | 1 | 1 | **Sexual precocity** | 16 | 0 | 1 |
| Liver abscess | 10 | 1 | 2 |  | 24 | 0 | 1 | **Stature** | 16 | 0 | 4 |
| Longissimus muscle area | 3 | 1 | 1 | **Milking speed** | 10 | 0 | 1 | **Stillbirth (maternal)** | 16 | 0 | 1 |
| Marbling score | 10 | 0 | 1 |  | 14 | 5 | 5 | **Strength** | 16 | 0 | 3 |
|  | 18 | 1 | 1 | **Muscle sodium content** | 14 | 1 | 1 | **Subcutaneous fat** | 16 | 0 | 1 |
|  | 29 | 1 | 1 | **Net merit** | 3 | 1 | 1 |  |  |  |  |
|  |  |  |  |  | 10 | 0 | 1 | **Teat placement - rear** | 16 | 0 | 2 |
|  | 9 | 1 | 1 |  | 16 | 0 | 1 |  |  |  |  |
|  |  |  |  |  | 17 | 1 | 0 |  |  |  |  |

**Table S7** Variance components for cow at weaning temperament using a repeatability model.

|  |  |  | **Highest Probability Density** | |
| --- | --- | --- | --- | --- |
| **Components** | **Mean** | **SD** | **Lower** | **Upper** |
| $\sigma_{CG}^{2}$ | 0.10 | 0.007 | 0.09 | 0.12 |
| $\sigma_{U}^{2}$ | 0.20 | 0.006 | 0.18 | 0.21 |
| $\sigma_{PE}^{2}$ | 0.02 | 0.003 | 0.017 | 0.03 |
| $\sigma_{e}^{2}$ | 0.12 | 0.008 | 0.11 | 0.14 |
| $h^{2}$ | 0.44 | 0.015 | 0.42 | 0.47 |
| *Rep* | 0.50 | 0.014 | 0.47 | 0.52 |
| *Threshold^1^* | 1.27 | 0.187 | 1.01 | 1.65 |

Results from a MCMC sampling from 200,000 iterations, 100,000 burn-in, and thinning of 10. ^1^Threshold is the third threshold value estimated to switch from score 3 to 4+; therefore, the liability distribution would have three thresholds: 0, 1, and the estimated as presented in this table. $\sigma_{CG}^{2}$: contemporary group variance; $\sigma_{U}^{2}$: additive genetic variance; $\sigma_{PE}^{2}$: permanent environment variance; $\sigma_{e}^{2}$: residual variance; $h^{2}$*:* heritability; Rep: repeatability; SD: standard deviation.

Model used: $l=et_{ct}+age_{days}+cg+u+pe+e$; temperament underlying a liability scale ($l$), conception type ($et_{ct}$), age at scoring in days ($age_{days}$), contemporary group ($cg$), additive genetic effect ($u$), permanent environment effect ($pe$), and residual ($e$).

**Table S8** (Co)variance components for yearling and cow at weaning temperament using a two-trait model.

|  |  |  | **Highest Probability Density** | |
| --- | --- | --- | --- | --- |
| **Components** | **Mean** | **SD** | **Lower** | **Upper** |
| YT $\sigma_{CG}^{2}$ | 0.16 | 0.008 | 0.15 | 0.18 |
| CT $\sigma_{CG}^{2}$ | 0.10 | 0.004 | 0.09 | 0.11 |
| YT $\sigma_{U}^{2}$ | 0.27 | 0.008 | 0.25 | 0.28 |
| YT and CT $\sigma_{u}$ | 0.19 | 0.006 | 0.18 | 0.21 |
| CT $\sigma_{U}^{2}$ | 0.20 | 0.006 | 0.19 | 0.21 |
| CT $\sigma_{PE}^{2}$ | 0.024 | 0.002 | 0.02 | 0.03 |
| YT $\sigma_{e}^{2}$ | 0.21 | 0.014 | 0.18 | 0.24 |
| CT $\sigma_{e}^{2}$ | 0.12 | 0.005 | 0.11 | 0.13 |
| YT threshold^1^ | 1.44 | 0.281 | 1.02 | 1.99 |
| CT threshold^1^ | 1.28 | 0.190 | 1.01 | 1.65 |
| YT $h^{2}$ | 0.42 | 0.011 | 0.40 | 0.44 |
| CT $h^{2}$ | 0.45 | 0.010 | 0.43 | 0.47 |
| CT *Rep* | 0.50 | 0.009 | 0.48 | 0.52 |
| YT and CT Genetic correlation | 0.84 | 0.009 | 0.83 | 0.86 |
| YT and CT Phenotypic correlation | 0.37 | 0.020 | 0.34 | 0.41 |

Results from a MCMC sampling from 50,000 iterations, 25,000 burn-in, and thinning of 10. ^1^Threshold is the third threshold value estimated to switch from score 3 to 4+; therefore, the liability distribution would have three thresholds: 0, 1, and the estimated as presented in this table. $\sigma_{CG}^{2}$: contemporary group variance; $\sigma_{U}^{2}$: additive genetic variance; $\sigma_{PE}^{2}$: permanent environment variance; $\sigma_{e}^{2}$: residual variance; $h^{2}$*:* heritability; Rep: repeatability; SD: standard deviation.

Model used: $\left[ \begin{matrix} l_{YT} \\ l_{CT} \end{matrix} \right]=\left[ \begin{matrix} et_{yt} \\ et_{ct} \end{matrix} \right]+\left[ \begin{matrix} calfagedev \\ age_{days} \end{matrix} \right]+\left[ \begin{matrix} aod \\ 0 \end{matrix} \right]+\left[ \begin{matrix} cg_{yt} \\ cg_{ct} \end{matrix} \right]+\left[ \begin{matrix} u_{yt} \\ u_{ct} \end{matrix} \right]+\left[ \begin{matrix} 0 \\ pe \end{matrix} \right]+\left[ \begin{matrix} e_{yt} \\ e_{ct} \end{matrix} \right]$; temperament underlying a liability scale ($l$), conception type ($et_{ct}$), age at scoring in days ($age_{days}$), contemporary group ($cg$), additive genetic effect ($u$), permanent environment effect ($pe$), and residual ($e$), YT is for yearling temperament (records on animals up to 460 days of age), and CT is cow at weaning temperament (records on cows older than 760 days of age).

**Table S9** (Co)variance components for yearling and cow at weaning temperament as a single-trait repeatability model.

|  |  |  | **Highest Probability Density** | |
| --- | --- | --- | --- | --- |
| **Components** | **Mean** | **SD** | **Lower** | **Upper** |
| $\sigma_{CG}^{2}$ | 0.13 | 0.01 | 0.11 | 0.15 |
| $\sigma_{U}^{2}$ | 0.24 | 0.01 | 0.22 | 0.26 |
| $\sigma_{PE}^{2}$ | 0.02 | 0.003 | 0.018 | 0.03 |
| $\sigma_{e}^{2}$ | 0.15 | 0.01 | 0.14 | 0.18 |
| Threshold | 1.36 | 0.24 | 1.01 | 1.83 |
| $h^{2}$ | 0.44 | 0.01 | 0.42 | 0.46 |
| Rep | 0.48 | 0.01 | 0.46 | 0.50 |

Results from a MCMC sampling from 200,000 iterations, 100,000 burn-in, and thinning of 10. ^1^Threshold is the third threshold value estimated to switch from score 3 to 4+; therefore, the liability distribution would have three thresholds: 0, 1, and the estimated as presented in this table. $\sigma_{CG}^{2}$: contemporary group variance; $\sigma_{U}^{2}$: additive genetic variance; $\sigma_{PE}^{2}$: permanent environment variance; $\sigma_{e}^{2}$: residual variance; $h^{2}$*:* heritability; Rep: repeatability; SD: standard deviation.

Model used: $l=et_{ct}+age_{days}+trait+aod+cg+u+pe+e$; temperament underlying a liability scale ($l$), conception type ($et_{ct}$), trait stage (i.e., yearling temperament or cow at weaning temperament, $trait$), age of the dam ($aod$), age at scoring in days ($age_{days}$), contemporary group ($cg$), additive genetic effect ($u$), permanent environment effect ($pe$), and residual ($e$).

**Table S10** (Co)variance components for average cow at weaning temperament (AverCT) and learning and behavioral plasticity (LBP) of a dataset containing animals with more than two records.

|  |  |  | **Highest Probability Density** | |
| --- | --- | --- | --- | --- |
| **Components** | **Mean** | **SD** | **Lower** | **Upper** |
| AverCT $\sigma_{CG}^{2}$ | 0.003 | 0.0006 | 0.002 | 0.0040 |
| AverCT and LBP $\sigma_{CG}$ | -0.002 | 0.0003 | -0.003 | -0.0018 |
| LBP $\sigma_{CG}^{2}$ | 0.002 | 0.0005 | 0.002 | 0.0035 |
| AverCT $\sigma_{U}^{2}$ | 0.246 | 0.0131 | 0.221 | 0.2691 |
| AverCT and LBP $\sigma_{u}$ | 0.030 | 0.0024 | 0.026 | 0.0345 |
| LBP $\sigma_{U}^{2}$ | 0.007 | 0.0006 | 0.006 | 0.0082 |
| AverCT $\sigma_{PE}^{2}$ | 0.066 | 0.0095 | 0.052 | 0.0849 |
| AverCT and LBP $\sigma_{pe}$ | 0.016 | 0.0034 | 0.011 | 0.0223 |
| LBP $\sigma_{PE}^{2}$ | 0.012 | 0.0018 | 0.009 | 0.0157 |
| $\sigma_{e}^{2}$ | 0.141 | 0.0110 | 0.122 | 0.1643 |
| AverCT $h^{2}$ | 0.472 | 0.0235 | 0.428 | 0.5123 |
| LBP $h^{2}$ | 0.014 | 0.0011 | 0.012 | 0.0159 |
| AverCT and LBP genetic correlation | 0.727 | 0.0298 | 0.689 | 0.7968 |
| AverCT *Rep* | 0.598 | 0.0125 | 0.572 | 0.6200 |
| LBP *Rep* | 0.038 | 0.0028 | 0.033 | 0.0434 |
| AverCT and LBP phenotypic correlation | 0.163 | 0.0103 | 0.144 | 0.1847 |

Results from a MCMC sampling from 50,000 iterations, 25,000 burn-in, and thinning of 10. $\sigma_{CG}^{2}$: contemporary group variance; $\sigma_{U}^{2}$: additive genetic variance; $\sigma_{PE}^{2}$: permanent environment variance; $\sigma_{e}^{2}$: residual variance; $h^{2}$*:* heritability; Rep: repeatability; SD: standard deviation.

**Table S11** Variance components over time for cow at weaning temperament using a random regression model and data of animals with at least two records.

|  | $\sigma_{CG}^{2}$ | | $\sigma_{U}^{2}$ | | $\sigma_{PE}^{2}$ | | $\sigma_{e}^{2}$ | | $h^{2}$ | | Rep | |
| --- | --- | --- | --- | --- | --- | --- | --- | --- | --- | --- | --- | --- |
| **Age** | **Mean** | **SD** | **Mean** | **SD** | **Mean** | **SD** | **Mean** | **SD** | **Mean** | **SD** | **Mean** | **SD** |
| 3 | 0.009 | 0.001 | 0.081 | 0.005 | 0.024 | 0.003 | 0.141 | 0.011 | 0.318 | 0.019 | 0.412 | 0.017 |
| 4 | 0.006 | 0.001 | 0.089 | 0.005 | 0.023 | 0.003 | 0.141 | 0.011 | 0.345 | 0.020 | 0.433 | 0.018 |
| 5 | 0.004 | 0.000 | 0.099 | 0.005 | 0.024 | 0.003 | 0.141 | 0.011 | 0.371 | 0.020 | 0.460 | 0.018 |
| 6 | 0.003 | 0.000 | 0.110 | 0.006 | 0.027 | 0.004 | 0.141 | 0.011 | 0.394 | 0.021 | 0.490 | 0.018 |
| 7 | 0.001 | 0.000 | 0.123 | 0.007 | 0.033 | 0.005 | 0.141 | 0.011 | 0.413 | 0.022 | 0.524 | 0.018 |
| 8 | 0.001 | 0.000 | 0.137 | 0.007 | 0.041 | 0.006 | 0.141 | 0.011 | 0.429 | 0.024 | 0.558 | 0.019 |
| 9 | 0.000 | 0.000 | 0.152 | 0.008 | 0.051 | 0.008 | 0.141 | 0.011 | 0.442 | 0.025 | 0.591 | 0.018 |
| 10 | 0.001 | 0.000 | 0.168 | 0.010 | 0.064 | 0.010 | 0.141 | 0.011 | 0.451 | 0.027 | 0.623 | 0.018 |
| >10 | 0.001 | 0.001 | 0.186 | 0.011 | 0.079 | 0.013 | 0.141 | 0.011 | 0.458 | 0.028 | 0.652 | 0.018 |

$\sigma_{CG}^{2}$: contemporary group variance; $\sigma_{U}^{2}$: additive genetic variance; $\sigma_{PE}^{2}$: permanent environment variance; $\sigma_{e}^{2}$: residual variance; $h^{2}$*:* heritability; Rep: repeatability; SD: standard deviation.
