# Supplementary material for: Unraveling the phenotypic and genomic background of behavioral plasticity and temperament in North American Angus cattle
Source: Genet Sel Evol. 2023 Jan 19;55:3. doi: 10.1186/s12711-023-00777-3 (PMC9850537; doi:10.1186/s12711-023-00777-3)

**Additional Figures**

**Figure S1** Manhattan plot for each age group of cows at weaning temperament (CT) considering the absolute effect of SNP-window.


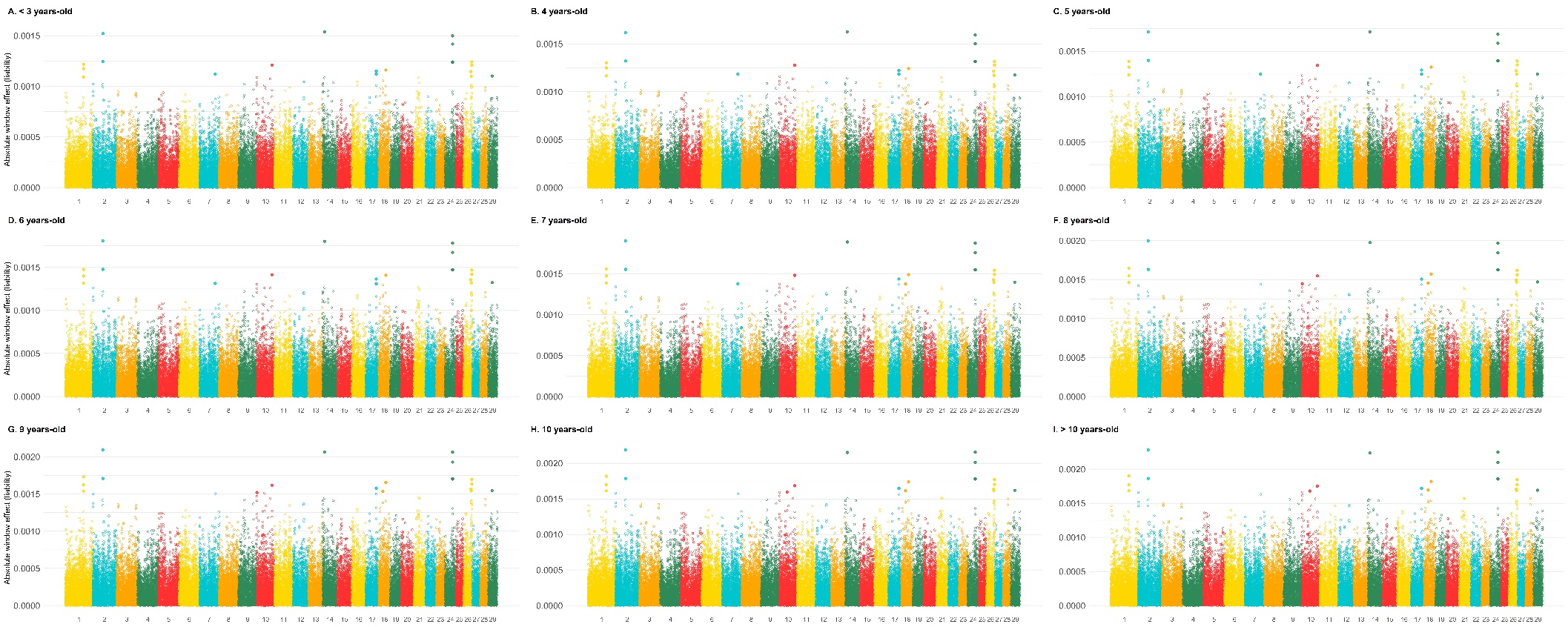

Supplement: Supplementary file 2 — Additional file 2: Figure S1. Manhattan plot for each age group of cows at weaning temperament (CT) considering the absolute effect of SNP-window. [file 12711_2023_777_MOESM2_ESM.docx]
